# Systemic pharmacological suppression of neural activity reverses learning impairment in a mouse model of Fragile X syndrome

**DOI:** 10.1101/2023.10.05.561013

**Published:** 2023-10-05

**Authors:** Amin Md Shakhawat, Jacqueline G Foltz, Adam B. Nance, Jaydev Bhateja, Jennifer L Raymond

**Affiliations:** 1 Department of Neurobiology, Stanford University, Stanford, California 94305-5125

## Abstract

The enhancement of associative synaptic plasticity often results in impaired rather than enhanced learning. Previously, we proposed that such learning impairments may result from saturation of the plasticity mechanism making it unavailable to be recruited at the appropriate synapses to support learning (Nguyen-Vu et al., 2017). This hypothesis was based on experimental results from mice lacking two class I major histocompatibility molecules, MHCI H2-K^b^ and H2-D^b^ (MHCI *K*^*b*^*D*^*b−/−*^), which have enhanced associative long-term depression at the parallel fiber-Purkinje cell synapses in the cerebellum (PF-Purkinje cell LTD). Here we extend this work by testing predictions of the saturation hypothesis in a second mouse line with enhanced PF-Purkinje cell LTD, the *Fmr1* knockout mouse model of Fragile X syndrome (FXS). Mice lacking *Fmr1* gene expression in cerebellar Purkinje cells (L7-*Fmr1* KO) were selectively impaired on an oculomotor learning task in which PF-Purkinje cell LTD has been implicated, with no impairment on an LTD-independent oculomotor learning task. Consistent with the saturation hypothesis, behavioral pre-training designed to reverse LTD at the PF-Purkinje cell synapses eliminated the oculomotor learning deficit in the L7-*Fmr1* KO mice, as previously reported in MHCI *K*^*b*^*D*^*b−/−*^mice. In addition, diazepam treatment to suppress neural activity and thereby limit the induction of associative LTD during the pre-training period also eliminated the learning deficit in L7-*Fmr1* KO mice. These results support the hypothesis that the enhancement of synaptic plasticity can lead to its saturation *in vivo* and inability to support learning, providing a novel mechanistic perspective that could inform the development of new clinical approaches for autism and other disorders of the nervous system.

## INTRODUCTION

Since its discovery, long term synaptic plasticity has been of great interest to neuroscientists as a therapeutic target for brain disorders, especially disorders affecting learning and memory. Scientific and technological advances have provided an array of tools for enhancing synaptic plasticity. In some cases, experimental manipulations that augment plasticity have succeeded in augmenting learning (Tang et al., 1999; Van Praag et al., 1999; Lee and Silva, 2009). However, in many cases, manipulations that augment plasticity have impaired learning (Migaud et al., 1998; Uetani et al., 2000; Gu et al., 2002; Cox et al., 2003; Hayashi et al., 2004; Rutten et al., 2011; Navakkode et al., 2022). Surprisingly, there have been few attempts to reconcile these conflicting findings with a mechanistic explanation for why enhancing synaptic plasticity can have opposite effects on learning. Such mechanistic insight about how enhanced synaptic plasticity functions *in vivo* could facilitate the development of this approach as a viable clinical intervention for learning disorders, recovery from stroke or brain injury, dementia, and other neurological and psychiatric disorders.

Recently, we proposed a testable hypothesis about what can go wrong with augmented plasticity *in vivo*, based on experimental and theoretical analysis of learning in mice with enhanced associative synaptic plasticity in the cerebellum (Nguyen-Vu et al., 2017). Associative LTD at the cerebellar PF-Purkinje cell synapses (PF-Purkinje cell LTD) has been implicated in certain cerebellum-dependent learning tasks and not others, based in part on the observation of selective learning impairments in mouse lines with impaired PF-Purkinje cell LTD (reviewed in Raymond and Medina, 2018; De Zeeuw et al., 2021). Initially, we expected that mice with enhanced PF-Purkinje cell LTD would exhibit the exact opposite behavioral phenotype as mice with impaired PF-Purkinje LTD, i.e., enhancement of learning on the same tasks in which mice with impaired PF-Purkinje cell LTD exhibit impaired learning. Contrary to this expectation, double knockout of the major histocompatibility class I molecules MHCI H2-K^b^ and H2-D^b^ (MHCI *K*^*b*^*D*^*b−/−*^), which enhances PF-Purkinje cell LTD (McConnell et al., 2009), results in the very same, specific oculomotor learning impairment as observed in mice with impaired PF-Purkinje cell LTD (Nguyen-Vu et al., 2017). To explain the puzzling observation that the enhancement of a plasticity mechanism could yield the same behavioral phenotype as its impairment, we hypothesized that enhanced LTD prevents learning by allowing spontaneous activity in the circuit to saturate this form of plasticity, making it unavailable at the specific synapses where it is needed to support learning. Two key predictions of the saturation hypothesis were confirmed experimentally by previous work: optogenetic stimulation of the circuit designed to saturate PF-Purkinje cell LTD before training recapitulated in WT mice the same, specific oculomotor learning deficit observed in the MHCI *K*^*b*^*D*^*b−/−*^ mice with enhanced LTD; and a behavioral manipulation designed to reverse PF-Purkinje cell LTD before oculomotor training reversed the learning deficit in MHCI *K*^*b*^*D*^*b−/−*^ mice (Nguyen-Vu et al., 2017).

Here we further tested the hypothesis that the enhancement of associative LTD at the parallel fiber-Purkinje cell synapses can result in the saturation of this plasticity mechanism before learning can take place and thereby impair learning. First, we replicated key behavioral findings in a different line of mice with enhanced LTD at the parallel fiber-Purkinje cell synapses. Purkinje cell-specific knock out of the Fragile X gene *Fmr1* enhances PF-Purkinje cell LTD (Koekkoek et al., 2005). We show that these L7-*Fmr1* KO mice are selectively impaired on an LTD-dependent oculomotor learning task, and that this learning deficit can be reversed with behavioral pre-training designed to reverse PF-Purkinje cell LTD, as previously reported in the MHCI *K*^*b*^*D*^*b−/−*^ mice with enhanced PF-Purkinje cell LTD. We then test a new prediction of the saturation hypothesis about a pharmacological treatment to reverse the learning deficit in mice with enhanced associative synaptic plasticity.

## RESULTS

### Selective learning impairment in mice with enhanced associative long-term depression in the cerebellum

We assessed oculomotor learning in mice lacking expression of the fragile X gene *Fmr1* in cerebellar Purkinje cells, which have been shown to have enhanced PF-Purkinje cell LTD (Koekkoek et al., 2005). Purkinje cell-specific *Fmr1* knock out mice were generated by crossing conditional *Fmr1* knockout mice (Mientjes et al., 2006) with mice expressing Cre under the control of the L7/Pcp2 promoter (Zhang et al., 2004; see Methods). We tested the ability of these L7-*Fmr1* KO mice to adaptively modify their vestibulo-ocular reflex (VOR), and compared their performance on different VOR learning tasks that have previously been shown to have different sensitivity to perturbations of PF-Purkinje cell LTD.

**Figure 1.**
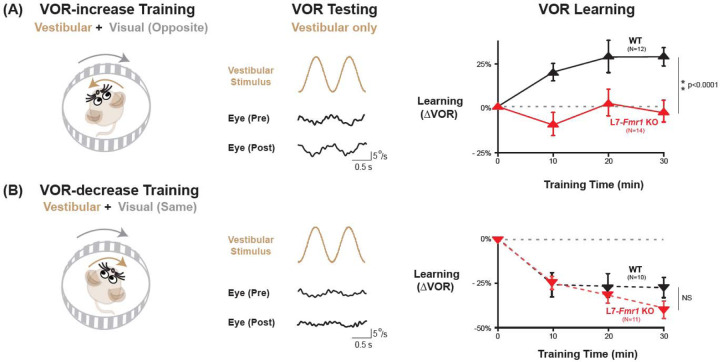
VOR-increase learning is impaired in L7-*Fmr1* KO mice with enhanced cerebellar LTD. **(A)** Training to increase the VOR. ***Left***, VOR-increase training paired a vestibular stimulus (1 Hz sinusoidal rotation about an earth-vertical axis, *brown*) with oppositely directed visual stimulus motion (*grey*). ***Middle***, Example raw eye velocity responses (*black*) to the vestibular stimulus alone in the dark, i.e., the VOR, measured Pre and Post VOR-increase training. ***Right***, Average learned change in the amplitude of the VOR relative to pre-training, measured in the dark (*upward triangles*) after each 10-min VOR-increase training block in the L7-*Fmr1* KO (*red*) and WT mice (*black*). **(B)** Training to decrease the VOR. ***Left***, VOR-decrease training paired a vestibular stimulus (1 Hz sinusoidal rotation) with visual stimulus motion in the same direction. ***Middle***, Example VOR responses in the dark, measured Pre and Post VOR-decrease training. ***Right***, VOR-decrease learning (*downward triangles*). NS= not significant. In this and all figures, values plotted are mean ± SEM.

The VOR stabilizes images on the retina by using the vestibular sensory input caused by a head movement to drive an oppositely directed eye movement response. Learning can adjust the amplitude of this oculomotor reflex to improve the stabilization of visual images on the retina for successful navigation in the world (Gonshor and Melvill Jones, 1973; Ito et al., 1974; Miles and Fuller, 1974; Gauthier and Robinson, 1975; Batini et al., 1979; Raymond, 1998; Broussard and Kassardjian, 2004; Gittis and du Lac, 2006; Cullen, 2023). Mice were trained to adaptively increase or decrease their VOR amplitude using two types of vestibular-visual stimulus pairings (Fig. 1; Boyden and Raymond 2003; Boyden et al., 2004). When a vestibular stimulus (1 Hz sinusoidal rotation about an earth-vertical axis with peak velocity of ±10°/s) was paired with oppositely directed motion of a large-field visual stimulus for 30 min (Fig. 1A, *left*; see Methods), this induced an adaptive learned increase in the eye movement responses of wild type (WT) mice to the vestibular stimulus alone (VOR-increase learning; Fig. 1A, *right, black*; p<0.001, 0 vs. 30 min, Tukey). When the vestibular stimulus was instead paired with motion of a visual stimulus in the same direction as the head (Fig. 1B, *left*), this induced an adaptive learned decrease in the eye movement responses of WT mice to the vestibular stimulus alone (VOR-decrease learning; Fig. 1B*, right, black;* p<0.001, 0 vs. 30 min, Tukey).

Both VOR-increase and VOR-decrease learning are cerebellum dependent (Ito et al., 1974; Robinson, 1976; Lisberger et al., 1984, 1994; Watanabe, 1984; Michnovicz and Bennett, 1987; Koekkoek et al., 1997; McElligott et al., 1998; Rambold et al., 2002). However, manipulations that impair or enhance PF-Purkinje cell LTD have previously been found to selectively alter VOR-increase learning, with less or no effect on VOR-decrease learning (Li et al., 1995; Boyden et al., 2006; Hansel et al., 2006; Guo et al., 2014; Kimpo et al., 2014; Nguyen-Vu et al., 2017; Zhang et al., 2023). Accordingly, the L7-*Fmr1* KO mice with enhanced PF-Purkinje cell LTD were selectively and profoundly impaired on VOR-increase learning. Unlike the WT control group, L7-*Fmr1* KO mice exhibited no significant change in the amplitude of their VOR after 30 min of VOR-increase training (Fig. 1A, *red*; p=0.97, L7-*Fmr1* KO, 0 vs 30 min; p<0.001, L7-*Fmr1* KO vs. WT, 30 min; Tukey). In contrast, VOR-decrease learning in the L7-*Fmr1* KO mice was robust and indistinguishable from that of their WT littermates (Fig. 1B*, red*; p<0.001, L7-*Fmr1* KO, 0 vs 30 min; p= 0.09, L7-*Fmr1* KO vs. WT; Tukey). Baseline oculomotor performance of L7-*Fmr1* KO mice was normal, as were the eye movement responses to the paired presentation of visual and vestibular stimuli used for both types of training (Fig. 1 - figure supplement 1), suggesting that there was no deficit in the vestibular, visual or oculomotor functions required to perform the learning tasks; rather the L7-*Fmr1* KO mice have a selective deficit in learning. These results support previous findings that manipulations of PF-Purkinje cell LTD selectively affect VOR-increase learning, and that the enhancement of PF-Purkinje cell LTD impairs rather than enhances this form of learning.

### Behavioral pre-training eliminates learning impairment in L7-*Fmr1* KO mice with enhanced LTD

A key question is why the enhancement of PF-Purkinje cell LTD would impair LTD-dependent learning. One potential explanation is that the enhancement of LTD allows the spontaneous activity in the cerebellar circuit to recruit and saturate this mechanism, making it unavailable to support new LTD-dependent learning. If this is the case, then manipulations that prevent or reverse excessive PF-Purkinje cell LTD before training should reset the circuit to a state compatible with new LTD-dependent learning, and thereby improve VOR-increase learning in the L7-*Fmr1* KO mice. To test this prediction, we first employed a behavioral approach designed to reverse PF-Purkinje cell LTD in the oculomotor cerebellum before training.

In wild-type mice, VOR-decrease training can rapidly reverse any behavioral evidence of prior VOR-increase learning, which suggests that VOR-decrease training can reverse any plasticity induced during VOR-increase learning, including any PF-Purkinje cell LTD (Boyden and Raymond, 2003). Accordingly, VOR-decrease pre-training was previously found to reverse the oculomotor learning deficit in *MHCI K*^*b*^*D*^*b−/−*^ mice with enhanced PF-Purkinje cell LTD (Nguyen-Vu et al., 2017). We tested whether the same behavioral pre-training intervention could also eliminate the learning deficient in L7-*Fmr1* KO mice.

**Figure 2.**
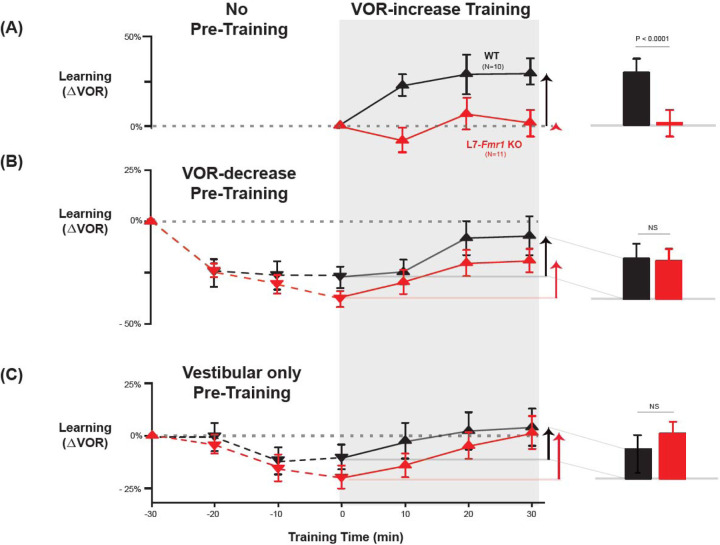
Behavioral pre-training rescued learning impairment of L7-*Fmr1* KO mice with enhanced associative LTD. Associative VOR-increase learning (*shaded area* and *bar graphs*), without pre-training (***A***), after VOR-decrease pre-training (***B***), and after Vestibular only pre-training (***C***). ***A,*** learned change in the VOR response measured in the dark after each 10-min block of VOR-increase training in the subset of L7-*Fmr1* KO (*red*) and WT (*black*) mice from Figure 1A that were also tested after pre-training. ***B,*** Changes in the VOR measured in the dark after each block of VOR-decrease pre-training *(downward triangles, dashed lines)* and then subsequent VOR-increase training *(upward triangles, solid lines)*. ***C*,** Changes in the VOR measured in the dark after each block of Vestibular only pre-training *(downward triangles, dashed lines)* and then VOR-increase training *(upward triangles, solid lines)*. ***Right,*** *Arrows* and *bars graphs* show the total change in the VOR induced by 30 min of VOR-increase training (training time = 30) compared with just before VOR-increase training (training time = 0).

L7-*Fmr1* KO and WT mice were subjected to 30 min of VOR-decrease pre-training followed by 30 min of VOR-increase training. In WT mice, there were adaptive changes in the amplitude of the VOR during both the pre-training and training periods—first a decrease and then an increase in the eye movement response to the vestibular stimulus alone (Fig. 2B, *black;* VOR-decrease, *dotted lines,* p<0.001, WT −30 vs 0 min; VOR-increase, *solid lines,* p=0.02, WT 0 vs 30 min; Tukey). The L7-*Fmr1* KO mice exhibited changes in the VOR during both the pre-training and training periods that were statistically indistinguishable from WT (Fig. 2B, *red;* VOR-decrease, *dotted lines,* p=0.18, L7-*Fmr1* KO vs. WT, 0 min, Tukey; Fig. 2B*, bar graphs,* p=0.17, L7-*Fmr1* KO vs. WT, VOR-increase from 0 to 30 min, t test; Fig. 2—figure supplement 1). Although in the absence of pre-training, VOR-increase training failed to induce any significant change in the VOR of the L7-*Fmr1* KO mice (Fig. 2A, *red;* p= 0.99, L7-*Fmr1* KO, 0 vs 30 min, Tukey), the same VOR-increase training procedure did induce a significant increase in VOR amplitude when delivered to the same cohort of mice after VOR-decrease pre-training (Fig. 2B, *red*, *solid lines and bar graph,* p<0.001, 0 vs 30 min, Tukey). In other words, the ability of the L7-*Fmr1* KO mice to learn in response to the VOR-increase training varied with the recent history of experience (Fig. 2, *compare red bars in* ***A*** *vs* ***B***; p< 0.05, VOR-increase learning of L7-*Fmr1* KO without vs. with pre-training, paired sample t-test). Pre-training experience did not have the same effect in WT mice. The amount of learning exhibited by WT mice in response to VOR-increase training was not enhanced after VOR-decrease pre-training (Fig. 2, *compare black bars in* ***A*** *vs* ***B***; p= 0.41, paired sample t-test). Thus, VOR-decrease pre-training had different effects on the L7-*Fmr1* KO and WT mice, putting the L7-*Fmr1* KO mice, but not the WT mice, into a state more compatible with VOR-increase learning.

A second behavioral pre-training procedure, habituation of the VOR, induced by presentation of the vestibular stimulus alone in complete darkness (Vestibular only pre-training), had effects similar to those of VOR-decrease pre-training on subsequent VOR-increase learning. After thirty minutes of Vestibular only pre-training, subsequent VOR-increase learning in the L7-*Fmr1* KO mice was comparable to that of their WT littermates (Fig. 2C, *red vs black bars;* p=0.84, L7-*Fmr1* KO vs. WT, VOR-increase from 0 to 30 min, paired sample t-test).

### Pharmacological suppression of neural activity the day before training eliminates learning impairment of L7-*Fmr1* KO mice with enhanced LTD

The preceding results are consistent with the hypothesis (Nguyen-Vu et al., 2017) that spontaneous activity in the circuit can induce and saturate PF-Purkinje cell LTD in mice with enhanced LTD, and that behavioral pre-training can alter neural activity in a manner that prevents or reverses this saturation, thereby reversing the learning impairment in mice with enhanced LTD. Since PF-Purkinje cell LTD is driven by co-activation of cerebellar parallel fibers and climbing fibers (Ito and Kano, 1982; Ito et al., 1982; Linden and Connor, 1995), pharmacological suppression of neural activity should also prevent the induction and saturation of LTD during the pre-training period, and restore the capacity for subsequent LTD-dependent learning in mice with enhanced LTD. We tested this prediction by administering the benzodiazepine diazepam, a positive allosteric modulator of GABA_A_ receptors, to enhance inhibition and suppress neural activity in the L7-*Fmr1* KO mice during the period preceding VOR-increase training. Diazepam has been shown to reduce neural firing in cerebellar neurons and neural responses to vestibular stimuli (Ryu and McCabe, 1974; Barmack, N. H. & Pettorossi, 1980). We assessed VOR learning 2 hours after diazepam administration, immediately after recovery from diazepam (18–24 hours after), and 1 week later.

The acute effect of diazepam administration was to impair learning. There was no effect of diazepam on the baseline amplitude of the VOR response measured in the dark 2 hours after diazepam (Fig. 3 – figure supplement 1), contrary to what has been reported in rabbit (Barmack, N. H. & Pettorossi, 1980). However, when VOR-increase training was delivered 2 hours after systemic administration of diazepam, VOR-increase learning was profoundly impaired in WT as well as L7-*Fmr1* KO mice (Fig. 3 – figure supplement 2).

**Figure 3.**
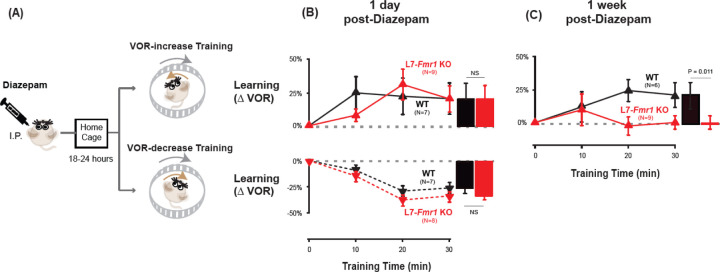
Diazepam pre-treatment rescued learning impairment of L7-*Fmr1* KO mice with enhanced associative LTD. **(A)** Mice were given an IP injection of diazepam (0.5 mg/kg) and then returned to the home cage for 18–24 hours, followed by VOR-increase (***top***) or VOR-decrease (***bottom***) training. **(B) *Top,*** VOR-increase learning 1 day (18–24 hours) after diazepam administration in L7-*Fmr1* KO (*red upward triangles*) and WT mice (*black upward triangles).* ***Bottom,*** VOR-decrease learning (*downward triangles)* 1 day after diazepam. **(C)** VOR-increase learning in the same mice as in **B**, 1 week after diazepam treatment, and 18–24 hours after IP saline injection.

It is not surprising that the acute effect of suppressing neural activity was to impair learning. The key question was whether this suppression of activity could reset the circuit to a state compatible with subsequent LTD-dependent learning. Therefore, VOR learning was tested after recovery from the acute effects of diazepam. Diazepam has a long half-life of ~24 hours (Riss et al., 2008), therefore mice were allowed to recover in their home cage for 18–24 hours after diazepam administration, and then VOR learning was tested after recovery from this prolonged period of pharmacological suppression of neural activity (Fig. 3A). Remarkably, the L7-*Fmr1* KO mice exhibited robust VOR-increase learning, comparable to their WT littermates (Fig. 3B, *top, red vs black*; p=0.86, Tukey). Although the same individual L7-*Fmr1* KO had exhibited no significant learning in response to VOR-increase training in the absence of the pharmacological pre-treatment (Fig. 2A, *red*; p= 0.99, 0 vs 30 min, Tukey), diazepam pre-treatment eliminated this learning deficit.

The enhancement of learning by diazepam pre-treatment was temporary. When the same mice were re-tested one week after diazepam administration, the L7-*Fmr1* KOs again failed to learn in response to VOR-increase training (Fig. 3C, *red;* p=0.12, 0 vs 30 min, Tukey). Thus, diazepam pre-treatment could restore the VOR circuit of L7-*Fmr1* KO mice to a state compatible with VOR-increase learning, but this effect was transient.

### Specificity of pre-training effects on learning

**Figure 4.**
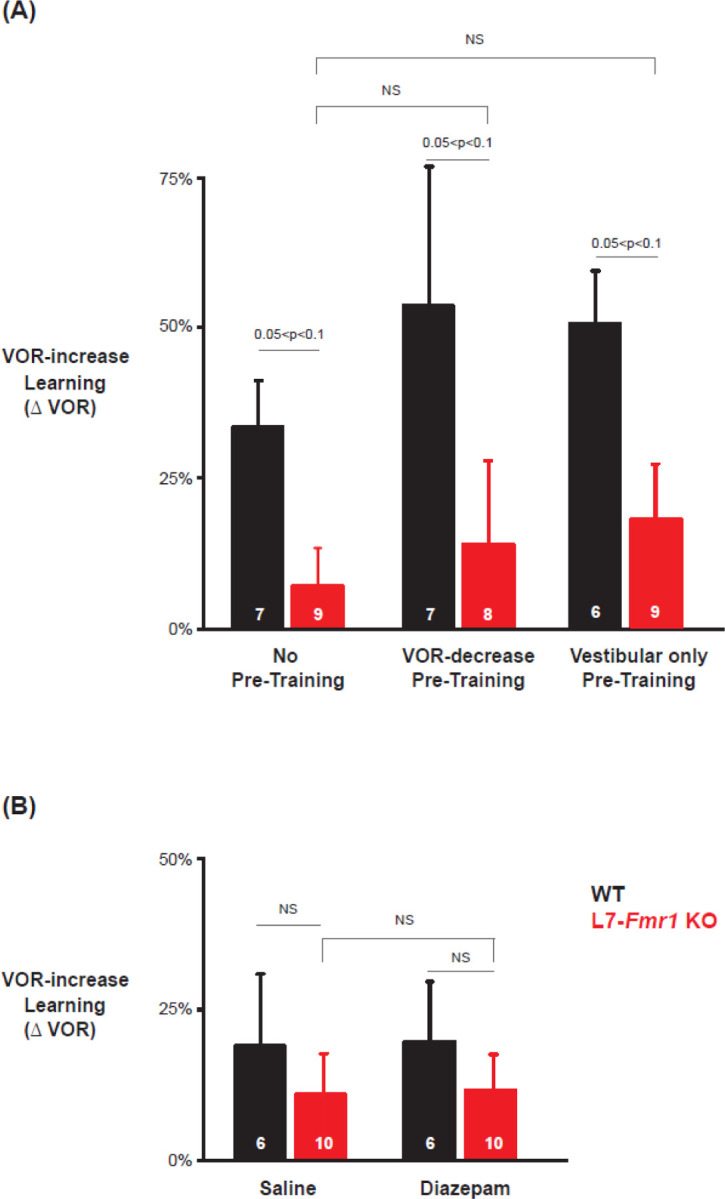
Low frequency (0.5 Hz) VOR-increase learning impairment was not rescued by behavioral pre-training or diazepam pre-treatment. **(A)** Low-frequency VOR-increase learning of L7-*Fmr1* KO mice (*red*) and WT mice (*black*), without pre-training (***left****)*, after 0.5 Hz VOR-decrease pre-training (***middle***), and after 0.5 Hz Vestibular only pre-training (***right***). **(B)** Low frequency (0.5 Hz) VOR-increase learning without diazepam pre-treatment (***left***) and 18–24 hours after IP injection of 0.5 mg/kg diazepam (***right***).

The ability of behavioral and pharmacological pre-training interventions to enhance learning was specific to mice with enhanced PF-Purkinje cell LTD and to the type of VOR learning task. Wild type mice did not exhibit enhanced VOR-increase learning after diazepam pre-treatment (Fig. 3B vs 3C, *top, black*; p= 0.55, paired sample t test). Moreover, there was no effect of diazepam pre-treatment on VOR-decrease learning in either the WT or L7-*Fmr1* KO mice (*compare* Fig. 3B, *bottom vs* 1B; p=0.11, L7-*Fmr1* KO vs. WT 1-day post-diazepam, VOR-decrease at 30 min, Tukey; p= 0.91, WT, diazepam vs control, VOR-decrease at 30 min paired sample t-test; p= 0.37, L7-*Fmr1* KO, diazepam vs control, VOR-decrease at 30 min, paired sample t-test). Thus, both the learning impairment in the L7-*Fmr1* KO mice and the effects of diazepam pre-treatment were selective for VOR-increase learning, consistent with previous evidence that this form of VOR learning is more dependent on PF-Purkinje cell LTD than VOR-decrease learning (Boyden et al., 2006; Nguyen-Vu et al., 2017). Previous work has also suggested a selective contribution of PF-Purkinje cell LTD to VOR learning induced with high-frequency (≥ 1 Hz) vestibular and visual stimuli, with less contribution of LTD when VOR learning is induced with low-frequency (≤0.66 Hz) vestibular and visual stimuli (Boyden et al., 2006; Nguyen Vu et al., 217). We found a trend for low-frequency (0.5 Hz) as well as high-frequency VOR-increase learning to be impaired in the L7-*Fmr1* KO mice (Fig. 4A, *left, red vs black;* p= 0.06, L7-*Fmr1* KO vs. WT, 30 min, Tukey). However, the low-frequency learning impairment was not reversed by the pre-training procedures that reversed the high-frequency learning impairment. Neither behavioral pre-training (Fig. 4A, *middle,* p= 0.47, VOR-decrease Pre-training vs. no Pre-training; Fig. 4A, *right,* p= 0.35, Vestibular only Pre-training vs. no Pre-training; paired sample t test), nor treatment with diazepam 18–24 hours before training (Fig. 4B, *compare red bars;* p= 0.66, saline vs. diazepam. paired sample t test), reversed the impairment of low-frequency VOR-increase learning in the L7-*Fmr1* KO mice, in contrast to their effectiveness at reversing the impairment of high-frequency VOR-increase learning (Figs. 2,3). This is consistent with the hypothesis that the behavioral and pharmacological pre-training manipulations selectively restore the capacity for learning tasks that depend on PF-Purkinje cell LTD.

## DISCUSSION

The results support the hypothesis proposed by Nguyen-Vu et al. (2017) that the enhancement of associative synaptic plasticity can impair learning by enabling ongoing neural activity in the circuit to saturate the plasticity mechanism. Consistent with this saturation hypothesis, L7-*Fmr1* KO mice with enhanced PF-Purkinje cell LTD exhibit impaired rather than enhanced learning on an oculomotor learning task in which PF-Purkinje cell LTD has been implicated, as previously shown for MHCI *K*^*b*^*D*^*b−/−*^ mice. Moreover, in both mouse lines, behavioral manipulations designed to prevent or reverse the saturation of PF-Purkinje cell LTD before training reversed the learning impairment, demonstrating that mice with enhanced LTD retain the capacity for robust LTD-dependent learning, although this capacity is influenced by the recent history of activity in the circuit. The new finding that pharmacological suppression of neural activity with diazepam can enhance subsequent learning in mice with enhanced associative plasticity provides new evidence for the saturation hypothesis and also could guide the development of novel clinical approaches.

The very similar behavioral phenotypes observed when LTD is enhanced by manipulating different molecular cascades strengthens the evidence that their shared effect of enhancing LTD is responsible for the learning impairment, rather than some other, off-target effect of the molecular manipulations. Whereas MHCI H2-*K*^*b*^ and H2-*D*^*b*^ act on MAP kinase and integrin via interaction with immune receptors such as PirB (Shatz, 2019), *Fmr1* acts by inhibiting mGluR-dependent dendritic protein translation (Huber et al., 2002). Previously, when different behavioral tasks were used to assess cerebellum-dependent learning in these two lines of mice, different phenotypes were reported–enhanced rotorod learning in the MHCI *K*^*b*^*D*^*b−/−*^ mice (McConnell et al., 2009), but impaired eyeblink conditioning in the L7-*Fmr1* KO mice, global *Fmr1* KO mice and Fragile X patients (Koekkoek et al., 2005). This highlights the importance of the specific choice of behavioral task for assessing cerebellar learning, and the differential dependence of different cerebellar learning tasks on specific molecular and cellular processes within the cerebellum.

Although PF-Purkinje cell LTD was the main candidate mechanism of cerebellum-dependent learning for many decades, there is growing evidence that PF-Purkinje cell LTD contributes selectively to certain cerebellum-dependent learning tasks, and not others (Shibuki et al., 1996; Van Alphen and De Zeeuw, 2002; Feil et al., 2003; Boyden et al., 2004; Boyden et al., 2006; Kimpo et al., 2014; Nguyen-Vu et al., 2017; De Zeeuw et al., 2021; Zhang et al., 2023). Oculomotor learning is particularly advantageous for studying the role of PF-Purkinje cell LTD in learning because this plasticity mechanism is thought to contribute differentially to closely related oculomotor learning tasks that all depend on the same sensory and motor signaling pathways through the cerebellar flocculus, providing powerful control conditions for distinguishing observations related to PF-Purkinje cell LTD vs. other functional components within the same circuit. The VOR learning tasks examined all involve a change in the gain of the eye movement response to a vestibular stimulus that is induced by pairing vestibular and visual stimuli. Despite these commonalities, a number of experimental approaches, including *ex vivo* slice physiology (Yamaguchi et al., 2016; Shim et al., 2022), optogenetic stimulation (Nguyen-Vu et al., 2017; Rowan et al., 2018; Zhang et al., 2023) and studies of oculomotor learning in mice with impaired LTD (Boyden et al., 2006; Hansel et al., 2006; Kakegawa et al., 2018) have suggested a selective contribution of PF-Purkinje cell LTD to VOR-increase learning induced by high-frequency (≥ 1 Hz) vestibular and visual stimuli, with less or no contribution to VOR-decrease learning or VOR-increase learning induced with lower frequency vestibular and visual stimuli. Accordingly, both lines of mice with enhanced LTD (L7-*Fmr1* KO and MHCI *K*^*b*^*D*^*b−/−*^ mice) exhibited selective alteration of VOR-increase and not VOR-decrease learning. In MHCI *K*^*b*^*D*^*b−/−*^ mice the impairment of VOR-increase learning was also selective for high-frequency training (Nguyen-Vu et al., 2017). In the L7-*Fmr1* KO mice, there was a trend for low-frequency as well as high-frequency VOR-increase learning to be impaired. However, the behavioral and diazepam pre-treatments designed to reverse the saturation of LTD only improved the high-frequency and not the low-frequency VOR-increase learning, suggesting different mechanistic underpinnings of the low- and high-frequency impairments. In other words, the deletion of *Fmr1* from Purkinje cells may have two distinct effects: enhancement of PF-Purkinje cell LTD, which recapitulates the high-frequency VOR-increase learning phenotypes observed in the MHCI *K*^*b*^*D*^*b−/−*^ mice with enhanced LTD, plus disruption of an additional cellular mechanism that contributes to low-frequency VOR-increase learning. Overall, in the two lines of mice with enhanced PF-Purkinje cell LTD, both the learning impairments and the effects of manipulations designed to reverse or prevent the saturation of LTD before training were remarkably selective for the specific VOR learning task in which PF-Purkinje cell LTD has been most strongly implicated.

**Figure 5.**
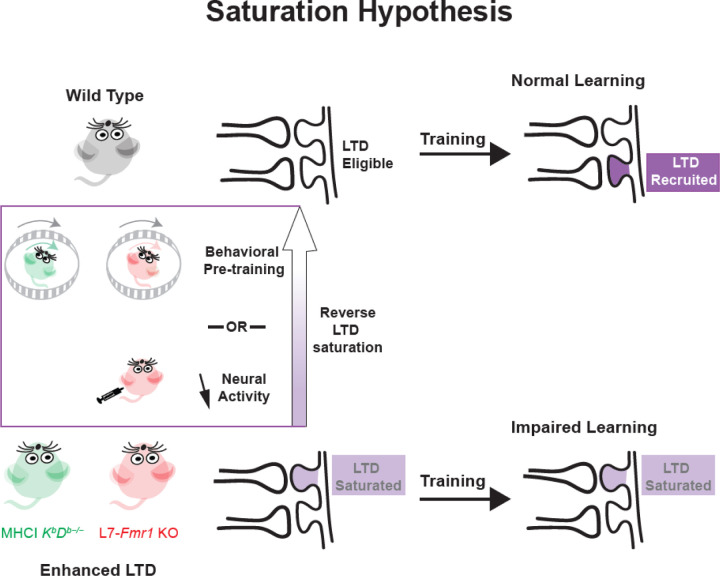
Saturation hypothesis for how enhanced plasticity could impair learning. ***Top,*** In naïve wild type mice, synapses are eligible to undergo associative synaptic plasticity (LTD; *dark violet*) in response to training, thereby supporting normal learning. ***Bottom,*** In mice with enhanced LTD, such as L7-*Fmr1* KO (*pink*) and MHCI *K*^*b*^*D*^*b−/−*^ (*green*), the lower threshold for induction of LTD allows it to be aberrantly recruited by spontaneous activity in the circuit (*light violet*), saturating the capacity for LTD and reducing its availability to be recruited during training at the synapses where it is needed to support learning, and thus impairing learning. Behavioral training that can reverse the LTD or drugs that reduce neural activity to reduce LTD induction can reset the synapses to an LTD-eligible state (*upward arrow*), restoring normal learning capacity.

A question of central scientific and clinical importance is why the enhancement of synaptic plasticity would impair rather than enhance learning. One hypothesis is that a lower threshold for LTD induction might cause it to be over-recruited during training, at synapses that should not have undergone LTD in addition to synapses where LTD would support adaptive behavioral changes, thereby corrupting the learning process (Migaud et al., 1998; Koekkoek et al., 2005; McConnell et al., 2009; Lee et al., 2014). Our alternative, saturation hypothesis suggests that the enhancement of PF-Purkinje cell LTD allows the spontaneous activity in the cerebellar circuit to recruit and saturate this mechanism even before training begins, making it unavailable during training to support new LTD-dependent learning (Fig. 5). Similarly, the aberrant recruitment of LTD before training may lead, not to its saturation *per se*, but to some other kind of reduced availability, such as an increased threshold for its induction (Bienenstock, Cooper, and Munro, 1982; Leet, Bear, and Gaier, 2022). This saturation or increased threshold hypothesis differs from the over-recruitment hypothesis by suggesting that LTD is under-rather than over-recruited during the training period in mice with enhanced LTD, and that the problem with enhanced LTD arises because of what it does to the circuit before training, rather than how it functions during training. Our finding that manipulations designed to prevent or reverse excessive PF-Purkinje cell LTD before training improve subsequent learning favor the saturation hypothesis.

Learning could be restored with two different approaches designed to prevent or reverse the saturation of LTD in the L7-*Fmr1* KO before training: behavioral pre-training (Fig. 2), and direct, pharmacological suppression of neural activity during the pre-training period (Fig. 3). In the absence of pre-training, high-frequency VOR-increase learning was not only impaired but completely absent in the L7-*Fmr1* KO mice (Fig. 1A, *right, red bar*). However, the same mice exhibited robust high-frequency VOR-increase learning, equal to that of WT, after behavioral pre-training, demonstrating that these mice can respond to the VOR-increase training with adaptive changes in the behavior (Fig. 2). At the end of those experiments, the amplitude of the VOR response (VOR gain = ratio of eye velocity to head velocity; see Methods) achieved in both L7-*Fmr1* KO and WT was roughly to the level of the VOR baseline value before pre-training. The additional experiments using pharmacological suppression of activity with diazepam during the pre-training period extends this work by providing the additional demonstration that the L7-*Fmr1* KO are capable of learning a VOR gain higher than baseline (Fig. 3). The ability of both behavioral and pharmacological pre-treatments to rescue learning shows that mice with enhanced LTD are not incapable of LTD-dependent learning. Rather, the enhancement of associative plasticity in the context of ongoing neural activity in the circuit appears to create a state in which LTD is saturated or otherwise unavailable to support learning. However, this saturated state can be reversed when the patterns of neural activity that create it are eliminated.

The striking specificity of the effects of the pre-training manipulations is consistent with the pre-training selectively reversing limitations caused by enhanced LTD, rather than generally enhancing cerebellum-dependent learning. Neither behavioral nor pharmacological pre-training enhanced learning in WT mice. Moreover, in the L7-*Fmr1* KO and MHCI *K*^*b*^*D*^*b−/−*^ mice, the pre-training treatments selectively enhanced high-frequency VOR-increase learning, with no significant effect on other forms of VOR learning (VOR-decrease learning or low-frequency VOR-increase learning), which previous research suggests are less dependent on PF-Purkinje cell LTD (Aiba et al., 1994; Li et al., 1995; Feil et al., 2003; Boyden et al., 2006; Hansel et al., 2006; Titley et al., 2010; Schonewille et al., 2011; Nguyen Vu et al., 2017; Zhang et al., 2023).

The present findings offer a scientific perspective that could guide the development of new clinical approaches for Fragile X syndrome and a range of other neurological and psychiatric conditions with enhanced associative plasticity. The oculomotor learning impairment in L7-*Fmr1* KO mice could be reversed with diazepam, which is an FDA-approved drug. The specificity of the effects of diazepam enhances its therapeutic potential. Pre-treatment with diazepam restored the capacity for high-frequency VOR-increase learning in the L7-*Fmr1* KO mice without compromising the normal performance of these mice on other oculomotor learning tasks. This was true even for VOR-decrease learning, which may depend on LTP of the same population of PF-Purkinje cell synapses that undergo LTD during VOR-increase learning (Shim et al., 2022). In other words, diazepam rescued the learning impairment with no apparent side effects on other, closely related functions of the same neural circuitry. At the same time, there is reason to expect that this approach of suppressing neural activity during a pre-training period may be generally applicable to any learning task, motor or cognitive, that is impaired by enhanced associative LTD (Rochefort et al., 2013; Badura et al., 2018; Ashburn et al., 2019; Frontera et al., 2020; Stoodley and Tsai, 2021; Hwang et al., 2022). Pharmacological suppression of neural activity should suppress PF-Purkinje cell LTD throughout the cerebellum, and hence may have the general effect of restoring all regions of the cerebellum to a state compatible with new LTD-dependent learning. This generality of the pharmacological approach stands in contrast to the behavioral pre-training approach, which would require extensive additional knowledge and experimentation to design the appropriate behavioral pre-treatment to reset each functional regional of the cerebellum to a state compatible with LTD-dependent learning, and different behavioral pre-treatments would be required to target each of the many functional regions supporting the myriad motor and cognitive functions of the cerebellum. Thus, from a practical standpoint, pharmacological pre-treatment to prevent or reverse the saturation of LTD before training is likely to be a more feasible and general approach to restoring the capacity for PF-Purkinje cell LTD-dependent learning. Alternative approaches for manipulating neural activity, such as transcranial magnetic stimulation (TMS) and transcranial direct current stimulation (tDCS), may also hold promise (Tan et al., 2013; Gschwind and Seeck, 2016; Biabani et al., 2017; Miterko et al., 2019; Denoyer et al., 2020).

## Conclusion

We leveraged the relatively simple and well understood physiology and function of the cerebellum and oculomotor system to develop and test a new hypothesis to explain why enhanced plasticity often impairs rather than enhances learning. The current results, along with the previous work by Nguyen-Vu and colleagues (2017) provide convergent evidence that a lower threshold for synaptic plasticity can result in its saturation and hence the impairment of learning. This saturation hypothesis may be useful in considering the impact of enhanced plasticity not only in the cerebellum, but in other brain areas as well. Moreover, the approach of limiting neural activity during a period before training may be broadly applicable for reversing maladaptive plasticity and resetting neural circuits to a state compatible with adaptive plasticity and new learning. For example, suppression of neural activity in the retina has been employed to reset the visual circuitry and enable recovery from amblyopia (Fong et al., 2021). The suppression of neural activity may be an especially useful approach if plasticity is pathologically enhanced in areas like the cerebellum or basal ganglia, with a high level of spontaneous spiking activity. More generally, the present results highlight the principle that synaptic properties do not control learning in isolation but interact with the patterns of neural activity in the corresponding circuits to control the capacity for new learning. The implication is that learning deficits associated with abnormal plasticity are not necessarily permanent, but in some cases can be remedied with appropriate reset of the circuit, opening up the possibility for therapeutic approaches targeting neural activity as well as the plasticity mechanisms themselves.

## Materials and Methods

All experimental procedures were approved by the Administrative Panel on Laboratory Animal Care at Stanford University.

### Mice

Mice with the *Fmr1* gene knocked out selectively from cerebellar Purkinje cells were generated through the following breeding strategy. First, homozygous female mice whose *Fmr1* gene, located on the X-chromosome, was floxed (*Fmr1* conditional knockout, cKO; Mientjes et al.., 2006) were crossed with male mice expressing L7/Pcp2-Cre on an autosome (L7/Pcp2-Cre *Jdhu*; The Jackson Laboratory, Stock No. 010536; Zhang et al., 2004;). The L7/Pcp2-Cre *Jdhu* line expresses Cre-recombinase in a manner that is highly selective for Purkinje cells. Male offspring from this first cross were mated with females homozygous for the *Fmr1 c*KO allele to generate offspring homozygous for *Fmr1* cKO, with some mice L7/Pcp2-Cre-positive and some L7/Pcp2-Cre-negative. Cre-positive offspring of this second cross are referred to as L7-*Fmr1* KO, and their Cre-negative littermates were used as controls and referred to as wild type (WT). Genotyping was performed by Transnetyx Inc on ear-clipped samples to confirm the presence of the floxed *Fmr1* allele in all mice and the presence or absence of Cre using RT-qPCR.

Mice were kept on a reversed 12-h light/12-h dark cycle, and behavioral experiments were conducted during the dark cycle of the mice. After surgical implantation (see below), mice were housed individually in standard cages and provided food and water *ad libidum.* Male and female mice 8–22 weeks old were used in the behavioral experiments. Similar learning deficits were observed in male and female L7-*Fmr1* KO mice (p=0.30, VOR-increase learning in 7 male vs 7 female L7-*Fmr1* KO mice, Fig. 1A), therefore results were pooled across sex.

### Surgery

Mice underwent surgery between 8–12 weeks of age to implant hardware for restraining the head and measuring eye movements, as described previously (Payne and Raymond, 2017; Nguyen-Vu et al., 2017). Mice were anesthetized with 1.5–2.5% isoflurane. An incision was made in the scalp and a custom-made head post (Shapeways Inc) was attached to the top of the skull using dental acrylic (Relyx Unicem Self-Adhesive Universal Resin Cement, Aplicap Capsule Refills-3M). Two stacked neodymium magnets with a total size of 0.75 × 2 mm (grade N50, axially magnetized, SuperMagnetMan.com) were implanted beneath the conjunctiva on the temporal side of the left eye. An angular magnetic field sensor (HMC1512, Honeywell Inc.) was soldered to an 8-pin connector and attached to the skull above the eye using dental acrylic, in a plane parallel to horizontal (nasal-temporal) eye movements. Eye movements were measured by detecting changes in the magnetic field created by movements of the magnet implanted on the eye (Payne and Raymond, 2017). Mice recovered from surgery for at least five days before experiments were performed.

### Behavioral experiments

Mice were acclimatized to the laboratory for at least 20 min after transport from the animal care facility before the start of an experiment. Experiments were conducted in a light-proof, sound-attenuated chamber (IAC acoustics). The head of the mouse was secured by attaching its head post to a restrainer, which was then attached to a vestibular turntable controlled by a Carco Model 823 rate table and Model 405D controller. The turntable delivered vestibular stimuli to the mouse by rotation about a yaw (earth-vertical) axis centered on the head of the mouse. An optokinetic drum controlled by a Yaskawa AC-Servo SGMCS-02B3B11 motor provided visual stimulation by rotation about an earth-vertical axis aligned with that of the vestibular turntable. The drum was made of translucent white plastic, and had alternating black and white stripes, with each stripe subtending approximately 7.5° of the visual field, illuminated by an LED light strip attached to the rim of the drum. Eye movements were recorded using the method described in Payne & Raymond (2017).

Experiments to assess VOR learning consisted of testing blocks and training blocks. Testing blocks consisted of three 45 second tests of the eye movement response to the vestibular stimulus delivered alone in complete darkness, i.e., the VOR. The vestibular stimulus was sinusoidal vestibular turntable rotation at 1 Hz or 0.5 Hz with a peak velocity of ±10°/s. The three 45 s VOR tests in a block were separated by 10 s with the turntable stationary. Training blocks were ten minutes long, and were repeated three times for a total of 30 min training, with a testing block following each training block. For VOR-increase training, the vestibular stimulus used for testing the VOR (1 Hz or 0.5 Hz) was paired with oppositely directed motion of the illuminated optokinetic drum with the same peak velocity (±10°/s). For VOR-decrease training, the vestibular stimulus used for testing was paired with motion of the optokinetic drum in the same direction with the same velocity, so that the drum was stationary relative to the head of the mouse. In behavioral pre-training experiments, the pre-training consisted of three 10-min blocks of either VOR-decrease training or delivery of the vestibular stimulus alone in the dark (Vestibular only), with a testing block before each training block. Calibration of the signals from the magnetic sensor used to record eye movements was performed after the experiment, as described in Payne & Raymond (2017).

Prior to some experiments (Figures 3, 3-figure supplement 1 and 2 and 4B,), mice received a single IP injection of 0.4, 0.5, or 2.5 mg/kg diazepam (in saline) or saline control. After diazepam or saline administration, mice were returned to the home cage, and then behavioral testing was performed either 2 hours, 18–24 hours, and/or 1 week later.

Each mouse underwent multiple behavioral experiments, with at least two days between successive experiments. The same cohort of mice was used for the experiments shown in Figures 1 and 2, with the order of the experiments randomized. A subset of the same cohort was then used for the diazepam experiments shown in Figure 3. A separate cohort of mice was used for the low-frequency training experiments shown in Figure 4, with the order of randomized for the behavioral pre-training conditions shown in Fig. 4A (no pretraining, VOR-decrease pre-training and Vestibular only pre-training) followed by the diazepam pre-treatment experiments in Fig. 4B, with randomized order for drug and saline conditions.

### Analysis of eye movement measurements

Signals from the magnetic sensor related to eye position were fourth-order low-pass (15 Hz) Butterworth filtered and then digitally differentiated to obtain eye velocity using a windowed (30 ms) Savitzky-Golay filter. Eye velocity data from each VOR test were fit with a 1 Hz or 0.5 Hz sinusoid. Values deviating from the sinusoidal fit by more than 31°/s were identified as saccades or movement artifacts and excluded, along with data from 50 ms before and after. Segments of data less than 10 ms in duration were also excluded. The entire 45 s VOR test was excluded if more than 45% of the data points were excluded. The amplitude of the sinusoidal fit provided the measure of the amplitude of the eye movement response, and values from the three VOR tests in a block were averaged. VOR learning (ΔVOR) was calculated as the percentage change in the VOR amplitude following each 10 min block of training relative to the baseline VOR amplitude measured before training. Eye movement gain was calculated at the ratio of eye movement amplitude to vestibular stimulus amplitude.

### Statistical analysis

Data were analyzed with a Shapiro-Wilk test of normality, followed by a two-factor repeated measures ANOVA with posthoc Tukey or by a two-sample or paired sample t-test, executed in OriginPro 2022 software. A value of p less than 0.05 was considered significant. Data are plotted as mean ± SEM.

### Code

All code used for data acquisition (https://github.com/RaymondLab/Code/tree/Master/Experiment%20Protocols) and analysis (https://github.com/RaymondLab/Code/tree/Master/Tools/VOR_Analysis) is available at github.com/RaymondLab/Code.

## Supplementary Material

Supplement 1

## References

[R1] Aiba A, Kano M, Chen C, Stanton ME, Fox GD, Herrup K, Zwingman TA, Tonegawa S (1994) Deficient cerebellar long-term depression and impaired motor learning in mGluR1 mutant mice. Cell 79:377–388. 7954803

[R2] Ashburn SM, Flowers DL, Napoliello EM, & Eden GF (2020). Cerebellar function in children with and without dyslexia during single word processing. Human Brain Mapping 41(1), 120–138 31597004 10.1002/hbm.24792 PMC7267899

[R3] Badura A, Verpeut JL, Metzger JW, Pereira TD, Pisano TJ, Deverett B, Bakshinskaya DE, & Wang S S (2018). Normal cognitive and social development require posterior cerebellar activity. ELife 7. 10.7554/eLife.36401 PMC6195348 30226467

[R4] Barmack NH (1980) Vestibulo-ocular Reflexes in Rabbits. Archives of Neurology 37:718. 7436816 10.1001/archneur.1980.00500600066014

[R5] Barmack NH, Pettorossi VE (1980) Influence of a GABA agonist, diazepam, on the vestibuloocular reflexes of the rabbit. Brain Research Bulletin 5:705–712.

[R6] Batini C, Ito M, Kado RT, Jastreboff PJ, Miyashita Y (1979) Interaction between the horizontal vestibulo ocular reflex and optokinetic response in rabbits. Experimental Brain Research 37:1–15. 314904 10.1007/BF01474249

[R7] Biabani M, Aminitehrani M, Zoghi M, Farrell M, Egan G, Jaberzadeh S (2017) The effects of transcranial direct current stimulation on short-interval intracortical inhibition and intracortical facilitation: a systematic review and meta-analysis. Reviews in the Neurosciences 29:99–114. 10.1515/revneuro-2017-0023 28820738

[R8] Bienenstock E, Cooper L, Munro P (1982) Theory for the development of neuron selectivity: orientation specificity and binocular interaction in visual cortex. The Journal of Neuroscience 2:32–48. 7054394 10.1523/JNEUROSCI.02-01-00032.1982 PMC6564292

[R9] Boyden ES, Katoh A, Pyle JL, Chatila TA, Tsien RW, Raymond JL (2006) Selective engagement of plasticity mechanisms for motor memory storage. Neuron 51:823–834. 16982426 10.1016/j.neuron.2006.08.026

[R10] Boyden ES, Katoh A, Raymond JL (2004) Cerebellum-dependent learning: the role of multiple plasticity mechanisms. Annual review of neuroscience 27:581–609. 10.1146/annurev.neuro.27.070203.144238 15217344

[R11] Boyden ES, Raymond JL (2003) Active Reversal of Motor Memories Reveals Rules Governing Memory Encoding. Neuron 39:1031–1042. 12971901 10.1016/s0896-6273(03)00562-2

[R12] Broussard DM, Kassardjian CD (2004) Learning in a Simple Motor System. Learning & Memory 11(2):127136. 10.1101/lm.65804 15054127

[R13] Cox PR, Fowler V, Xu B, Sweatt JD, Paylor R, Zoghbi HY (2003) Mice lacking tropomodulin-2 show enhanced long-term potentiation, hyperactivity, and deficits in learning and memory. Molecular and Cellular Neuroscience 23:1–12. 12799133 10.1016/s1044-7431(03)00025-3

[R14] Cullen KE (2023). Vestibular motor control. Handbook of clinical Neurology. 195: 31–54. 37562876 10.1016/B978-0-323-98818-6.00022-4

[R15] Denoyer Y, Merlet I, Wendling F, Benquet P (2020) Modelling acute and lasting effects of tDCS on epileptic activity. Journal of Computational Neuroscience 48:161–176. 32307640 10.1007/s10827-020-00745-6

[R16] De Zeeuw CI, Lisberger SG, Raymond JL (2021) Diversity and dynamism in the cerebellum. Nature Neuroscience 24:160–167. 33288911 10.1038/s41593-020-00754-9

[R17] Dworkin JD, Linn KA, Teich EG, Zurn P, Shinohara RT, Bassett DS (2020) The extent and drivers of gender imbalance in neuroscience reference lists. Nature Neuroscience 23 (8): 918–926. 32561883 10.1038/s41593-020-0658-y

[R18] Feil R, Hartmann J, Luo C, Wolfsgruber W, Schilling K, Feil S, Barski JJ, Meyer M, Konnerth A, De Zeeuw CI, Hofmann F (2003) Impairment of LTD and cerebellar learning by Purkinje cell–specific ablation of cGMP-dependent protein kinase I. Journal of Cell Biology 163:295–302. 14568994 10.1083/jcb.200306148 PMC2173527

[R19] Fong M, Duffy KR, Leet MP, Candler CT, Bear MF (2021) Correction of amblyopia in cats and mice after the critical period. eLife 10. 10.7554/eLife.70023 PMC8456712 34464258

[R20] Frontera JL, Baba Aissa H, Sala RW, Mailhes-Hamon C, Georgescu IA, Léna C, & Popa D (2020). Bidirectional control of fear memories by cerebellar neurons projecting to the ventrolateral periaqueductal grey. Nature Communications 11(1), 5207. 10.1038/s41467-020-18953-0 PMC7566591 33060630

[R21] Gauthier GM, Robinson DA (1975) Adaptation of the human vestibuloocular reflex to magnifying lenses. Brain Research 92:331–335. 1080685 10.1016/0006-8993(75)90279-6

[R22] Gittis AH & de Lac S (2006) Intrinsic and synaptic plasticity in the vestibular system. Current Opinion in Neurobiology 16(4), 385–390. 16842990 10.1016/j.conb.2006.06.012

[R23] Gonshor A, Jones GM (1973) Proceedings: Changes of human vestibulo-ocular response induced by vision-reversal during head rotation. The Journal of physiology 234:102P–103P. 4544793

[R24] Gschwind M, Seeck M (2016) Transcranial direct-current stimulation as treatment in epilepsy. Expert Review of Neurotherapeutics 16:1427–1441. 27384886 10.1080/14737175.2016.1209410

[R25] Guo CC, Ke MC, Raymond JL (2014) Cerebellar Encoding of Multiple Candidate Error Cues in the Service of Motor Learning. Journal of Neuroscience 34:9880–9890. 25057191 10.1523/JNEUROSCI.5114-13.2014 PMC4107405

[R26] Gu Y, McIlwain KL, Weeber EJ, Yamagata T, Xu B, Antalffy BA, Reyes C, Yuva-Paylor L, Armstrong D, Zoghbi H, Sweatt JD, Paylor R, Nelson DL (2002) Impaired Conditioned Fear and Enhanced Long Term Potentiation in Fmr2 Knock-Out Mice. The Journal of Neuroscience 22:2753–2763. 11923441 10.1523/JNEUROSCI.22-07-02753.2002 PMC6758318

[R27] Hansel C, de Jeu M, Belmeguenai A, Houtman SH, Buitendijk GHS, Andreev D, De Zeeuw CI, Elgersma Y (2006) alphaCaMKII Is essential for cerebellar LTD and motor learning. Neuron 51:835–843. 16982427 10.1016/j.neuron.2006.08.013

[R28] Hayashi ML, Choi S-Y, Rao BSS, Jung H-Y, Lee H-K, Zhang D, Chattarji S, Kirkwood A, Tonegawa S (2004) Altered Cortical Synaptic Morphology and Impaired Memory Consolidation in Forebrain-Specific Dominant-Negative PAK Transgenic Mice. Neuron 42:773–787. 15182717 10.1016/j.neuron.2004.05.003

[R29] Huber KM, Gallagher SM, Warren ST, Bear MF (2002) Altered synaptic plasticity in a mouse model of fragile X mental retardation. Proceedings of the National Academy of Sciences 99:7746–7750. 10.1073/pnas.122205699 PMC124340 12032354

[R30] Hwang KD, Kim SJ, & Lee YS (2022). Cerebellar Circuits for Classical Fear Conditioning. Frontiers in Cellular Neuroscience, 16. 10.3389/fncel.2022.836948 PMC9005982 35431810

[R31] Ito M, Kano M (1982) Long-lasting depression of parallel fiber-Purkinje cell transmission induced by conjunctive stimulation of parallel fibers and climbing fibers in the cerebellar cortex. Neuroscience Letters 33:253–258. 6298664 10.1016/0304-3940(82)90380-9

[R32] Ito M, Sakurai M, Tongroach P (1982) Climbing fibre induced depression of both mossy fibre responsiveness and glutamate sensitivity of cerebellar Purkinje cells. The Journal of physiology 324:113–134. 7097592 10.1113/jphysiol.1982.sp014103 PMC1250696

[R33] Ito M, Shiida T, Yagi N, Yamamoto M (1974) The Cerebellar Modification of Rabbit’s Horizontal Vestibulo-Ocular Reflex Induced by Sustained Head Rotation Combined with Visual Stimulation. Proceedings of the Japan Academy 50:85–89.

[R34] Ito M, Shiida T, Yagi N, Yamamoto M (1974) Visual influence on rabbit horizontal vestibulo-ocular reflex presumably effected via the cerebellar flocculus. Brain Research 65:170–174. 4810171 10.1016/0006-8993(74)90344-8

[R35] Kakegawa W, Katoh A, Narumi S, Miura E, Motohashi J, Takahashi A, Kohda K, Fukazawa Y, Yuzaki M, Matsuda S (2018) Optogenetic Control of Synaptic AMPA Receptor Endocytosis Reveals Roles of LTD in Motor Learning. Neuron 99:985–998.e6. 30122381 10.1016/j.neuron.2018.07.034

[R36] Kimpo RR, Rinaldi JM, Kim CK, Payne HL, Raymond JL (2014) Gating of neural error signals during motor learning. eLife 3. 10.7554/eLife.02076 PMC3989583 24755290

[R37] Koekkoek SKE (2005) Deletion of FMR1 in Purkinje Cells Enhances Parallel Fiber LTD, Enlarges Spines, and Attenuates Cerebellar Eyelid Conditioning in Fragile X Syndrome. Neuron 47:339–352. 16055059 10.1016/j.neuron.2005.07.005

[R38] Koekkoek S, V. Alphen A, V.d. Burg J, Grosveld F, Galjart N, De Zeeuw C (1997) Gain adaptation and phase dynamics of compensatory eye movements in mice. Genes and Function 1:175–190. 9680293 10.1046/j.1365-4624.1997.00018.x

[R39] Leet MP, Bear MF, Gaier ED (2022) Metaplasticity: a key to visual recovery from amblyopia in adulthood? Current Opinion in Ophthalmology 33:512–518. 36094025 10.1097/ICU.0000000000000901 PMC9547850

[R40] Lee Y-S, Silva AJ (2009) The molecular and cellular biology of enhanced cognition. Nature Reviews Neuroscience 10:126–140. 19153576 10.1038/nrn2572 PMC2664745

[R41] Lee H, Brott BK, Kirkby LA, Adelson JD, Cheng S, Feller MB, Datwani A, & Shatz CJ (2014). Synapse elimination and learning rules co-regulated by MHC class I H2-Db. Nature 509(7499), 195–200. 24695230 10.1038/nature13154 PMC4016165

[R42] Li J, Smith SS, McElligott JG (1995) Cerebellar nitric oxide is necessary for vestibulo-ocular reflex adaptation, a sensorimotor model of learning. Journal of Neurophysiology 74:489–494. 7472353 10.1152/jn.1995.74.1.489

[R43] Linden DJ, Connor JA (1995) Long-term synaptic depression. Annual review of neuroscience 18:319–357. 10.1146/annurev.ne.18.030195.001535 7605065

[R44] Lisberger SG, Miles FA, Zee DS (1984) Signals used to compute errors in monkey vestibuloocular reflex: possible role of flocculus. Journal of Neurophysiology 52:1140–1153. 6335171 10.1152/jn.1984.52.6.1140

[R45] Lisberger SG, Pavelko TA, Bronte-Stewart HM, Stone LS (1994) Neural basis for motor learning in the vestibuloocular reflex of primates. II. Changes in the responses of horizontal gaze velocity Purkinje cells in the cerebellar flocculus and ventral paraflocculus. Journal of Neurophysiology 72:954–973. 7983548 10.1152/jn.1994.72.2.954

[R46] McConnell MJ, Huang YH, Datwani A, Shatz CJ (2009) H2-Kb and H2-Db regulate cerebellar long-term depression and limit motor learning. Proceedings of the National Academy of Sciences 106:6784–6789. 10.1073/pnas.0902018106 PMC2672503 19346486

[R47] McElligott JG, Beeton P, Polk J (1998) Effect of Cerebellar Inactivation by Lidocaine Microdialysis on the Vestibuloocular Reflex in Goldfish. J Neurophysiol 79:1286–1294. 9497410 10.1152/jn.1998.79.3.1286

[R48] Michnovicz JJ, Bennett MVL (1987) Effects of rapid cerebellectomy on adaptive gain control of the vestibulo-ocular reflex in alert goldfish. Experimental Brain Research 66. 10.1007/BF00243305 3595775

[R49] Mientjes EJ, Nieuwenhuizen I, Kirkpatrick L, Zu T, Hoogeveen-Westerveld M, Severijnen L, Rifé M, Willemsen R, Nelson DL, Oostra BA (2006) The generation of a conditional Fmr1 knock out mouse model to study Fmrp function in vivo. Neurobiology of Disease 21:549–555. 16257225 10.1016/j.nbd.2005.08.019

[R50] Migaud M, Charlesworth P, Dempster M, Webster LC, Watabe AM, Makhinson M, He Y, Ramsay MF, Morris RGM, Morrison JH, O’Dell TJ, Grant SGN (1998) Enhanced long-term potentiation and impaired learning in mice with mutant postsynaptic density-95 protein. Nature 396:433–439. 9853749 10.1038/24790

[R51] Miles FA, Fuller JH (1974) Adaptive plasticity in the vestibulo-ocular responses of the rhesus monkey. Brain Research 80:512–516. 4547632 10.1016/0006-8993(74)91035-x

[R52] Miterko LN, Baker KB, Beckinghausen J, Bradnam LV, Cheng MY, Cooperrider J, DeLong MR, Gornati SV, Hallett M, Heck DH, Hoebeek FE, Kouzani AZ, Kuo S-H, Louis ED, Machado A, Manto M, McCambridge AB, Nitsche MA, Taib NO Ben, … Sillitoe RV(2019). Consensus Paper: Experimental Neurostimulation of the Cerebellum. The Cerebellum 18(6), 1064–1097. 31165428 10.1007/s12311-019-01041-5 PMC6867990

[R53] Navakkode S, Zhai J, Wong YP, Li G, Soong TW (2022) Enhanced long-term potentiation and impaired learning in mice lacking alternative exon 33 of CaV1.2 calcium channel. Translational Psychiatry 12:1. 35013113 10.1038/s41398-021-01683-2 PMC8748671

[R54] Nguyen-Vu TDB, Zhao GQ, Lahiri S, Kimpo RR, Lee H, Ganguli S, Shatz CJ, Raymond JL (2017) A saturation hypothesis to explain both enhanced and impaired learning with enhanced plasticity. eLife 6. 10.7554/eLife.20147 PMC5386593 28234229

[R55] Payne HL, Raymond JL (2017) Magnetic eye tracking in mice. eLife 6. 10.7554/eLife.29222 PMC5584990 28872455

[R56] Rambold H, Churchland A, Selig Y, Jasmin L, Lisberger SG (2002) Partial Ablations of the Flocculus and Ventral Paraflocculus in Monkeys Cause Linked Deficits in Smooth Pursuit Eye Movements and Adaptive Modification of the VOR. Journal of Neurophysiology 87:912–924. 11826056 10.1152/jn.00768.2000 PMC2629758

[R57] Raymond JL (1998) Learning in the oculomotor system: from molecules to behavior. Curr Opin Neurobiol 8:770–776. 9914237 10.1016/s0959-4388(98)80120-7

[R58] Raymond JL, Medina JF (2018) Computational Principles of Supervised Learning in the Cerebellum. Annual Review of Neuroscience 41:233–253. 10.1146/annurev-neuro-080317-061948 PMC6056176 29986160

[R59] Riss J, Cloyd J, Gates J, Collins S (2008) Benzodiazepines in epilepsy: pharmacology and pharmacokinetics. Acta Neurologica Scandinavica 118:69–86. 18384456 10.1111/j.1600-0404.2008.01004.x

[R60] Robinson DA (1976) Adaptive gain control of vestibuloocular reflex by the cerebellum. Journal of Neurophysiology 39:954–969. 1086347 10.1152/jn.1976.39.5.954

[R61] Rochefort C, Lefort J, & Rondi-Reig L (2013). The cerebellum: a new key structure in the navigation system. Frontiers in Neural Circuits 7. 10.3389/fncir.2013.00035 PMC3595517 23493515

[R62] Rowan MJM, Bonnan A, Zhang K, Amat SB, Kikuchi C, Taniguchi H, Augustine GJ, Christie JM (2018) Graded Control of Climbing-Fiber-Mediated Plasticity and Learning by Inhibition in the Cerebellum. Neuron 99:999–1015.e6 30122378 10.1016/j.neuron.2018.07.024 PMC6206434

[R63] Rutten K, Wallace TL, Works M, Prickaerts J, Blokland A, Novak TJ, Santarelli L, Misner DL (2011) Enhanced long-term depression and impaired reversal learning in phosphodiesterase 4B-knockout (PDE4B−/−) mice. Neuropharmacology 61:138–147. 21458469 10.1016/j.neuropharm.2011.03.020

[R64] Ryu JH, McCabe BF (1974) effects of diazepam and dimenhydrinate on the resting activity of the vestibular neuron. Aerospace medicine 45:1177–1179. 4429059

[R65] Schonewille M, Gao Z, Boele H-J, Vinueza Veloz MF, Amerika WE, Šimek AAM, De Jeu MT, Steinberg JP, Takamiya K, Hoebeek FE, Linden DJ, Huganir RL, De Zeeuw CI (2011) Reevaluating the Role of LTD in Cerebellar Motor Learning. Neuron 70:43–50. 21482355 10.1016/j.neuron.2011.02.044 PMC3104468

[R66] Shatz CJ (2009) MHC Class I: An Unexpected Role in Neuronal Plasticity. Neuron 64:40–45. 19840547 10.1016/j.neuron.2009.09.044 PMC2773547

[R67] Shibuki K, Gomi H, Chen L, Bao S, Kim JJ, Wakatsuki H, Fujisaki T, Fujimoto K, Katoh A, Ikeda T, Chen C, Thompson RF, Itohara S (1996) Deficient Cerebellar Long-Term Depression, Impaired Eyeblink Conditioning, and Normal Motor Coordination in GFAP Mutant Mice. Neuron 16:587–599. 8785056 10.1016/s0896-6273(00)80078-1

[R68] Shim H., Fanning A., Raymond J. (2022). Plasticity mechanisms for bidirectional oculomotor learning. Program No. 213.05. 2022 Neuroscience Meeting Planner. San Diego, CA: Society for Neuroscience, 2022. Online.

[R69] Stoodley CJ, & Tsai PT (2021). Adaptive Prediction for Social Contexts: The Cerebellar Contribution to Typical and Atypical Social Behaviors. Annual Review of Neuroscience 44(1), 475–493. 10.1146/annurev-neuro-100120-092143 PMC9037460 34236892

[R70] Tang Y-P, Shimizu E, Dube GR, Rampon C, Kerchner GA, Zhuo M, Liu G, Tsien JZ (1999) Genetic enhancement of learning and memory in mice. Nature 401:63–69. 10485705 10.1038/43432

[R71] Tan T, Xie J, Tong Z, Liu T, Chen X, Tian X (2013) Repetitive transcranial magnetic stimulation increases excitability of hippocampal CA1 pyramidal neurons. Brain Research 1520:23–35. 23651978 10.1016/j.brainres.2013.04.053

[R72] Titley HK, Heskin-Sweezie R, Broussard DM (2010) The Bidirectionality of Motor Learning in the Vestibulo-ocular Reflex Is a Function of Cerebellar mGluR1 Receptors. Journal of Neurophysiology 104:3657–3666. 20926606 10.1152/jn.00664.2010

[R73] Uetani N (2000) Impaired learning with enhanced hippocampal long-term potentiation in PTPdelta deficient mice. The EMBO Journal 19:2775–2785. 10856223 10.1093/emboj/19.12.2775 PMC203365

[R74] Van Alphen AM, De Zeeuw CI (2002) Cerebellar LTD facilitates but is not essential for long-term adaptation of the vestibulo-ocular reflex. In: European Journal of Neuroscience 16 (3): 486–490. 12193192 10.1046/j.1460-9568.2002.02094.x

[R75] Van Praag H, Christie BR, Sejnowski TJ, Gage FH (1999) Running enhances neurogenesis, learning, and long-term potentiation in mice. Proceedings of the National Academy of Sciences 96:13427–13431. 10.1073/pnas.96.23.13427 PMC23964 10557337

[R76] Watanabe E (1984) Neuronal events correlated with long-term adaptation of the horizontal vestibulo ocular reflex in the primate flocculus. Brain Research 297:169–174. 6609741 10.1016/0006-8993(84)90555-9

[R77] Witter L, Rudolph S, Pressler RT, Lahlaf SI, Regehr WG (2016) Purkinje Cell Collaterals Enable Output Signals from the Cerebellar Cortex to Feed Back to Purkinje Cells and Interneurons. Neuron 91:312–319. 27346533 10.1016/j.neuron.2016.05.037 PMC4969194

[R78] Yamaguchi K, Itohara S, Ito M (2016) Reassessment of long-term depression in cerebellar Purkinje cells in mice carrying mutated GluA2 C terminus. Proceedings of the National Academy of Sciences 113:10192–10197. 10.1073/pnas.1609957113 PMC5018784 27551099

[R79] Zhang X-M, Ng AH-L, Tanner JA, Wu W-T, Copeland NG, Jenkins NA, Huang J-D (2004) Highly restricted expression of Cre recombinase in cerebellar Purkinje cells. Genesis 40:45–51. 15354293 10.1002/gene.20062

[R80] Zhang K, Yang Z, Gaffield MA, Gross GG, & Arnold DB, Christie JM (2023). Molecular layer disinhibition unlocks climbing-fiber-instructed motor learning in the cerebellum. bioRxiv 2023-08.

[R81] Zhou D., Cornblath EJ, Stiso J, Teich EG, Dworkin JD, Blevins AS, & Bassett DS(2020). Gender diversity statement and code notebook v1. 0. Zenodo.

[R82] Zurn P, Bassett DS, & Rust NC (2020). The Citation Diversity Statement: A Practice of Transparency, A Way of Life. Trends in Cognitive Sciences 24(9), 669–672. 32762966 10.1016/j.tics.2020.06.009

