# Supplementary material for: Systemic pharmacological suppression of neural activity reverses learning impairment in a mouse model of Fragile X syndrome
Source: bioRxiv. 2024 Apr 5:2023.10.05.561013. Originally published 2023 Oct 5. Preprint. [Version 2] doi: 10.1101/2023.10.05.561013 (PMC10592955; doi:10.1101/2023.10.05.561013)
Supplement: 1 [file NIHPP2023.10.05.561013V2-supplement-1.pdf]

bioRxiv preprint doi: https://doi.org/10.1101/2023.10.05.561013; this version posted April 5, 2024. The copyright holder for this preprint (which was not certified by peer review) is the author/funder, who has granted bioRxiv a license to display the preprint in perpetuity. It is made available under aCC-BY 4.0 International license.

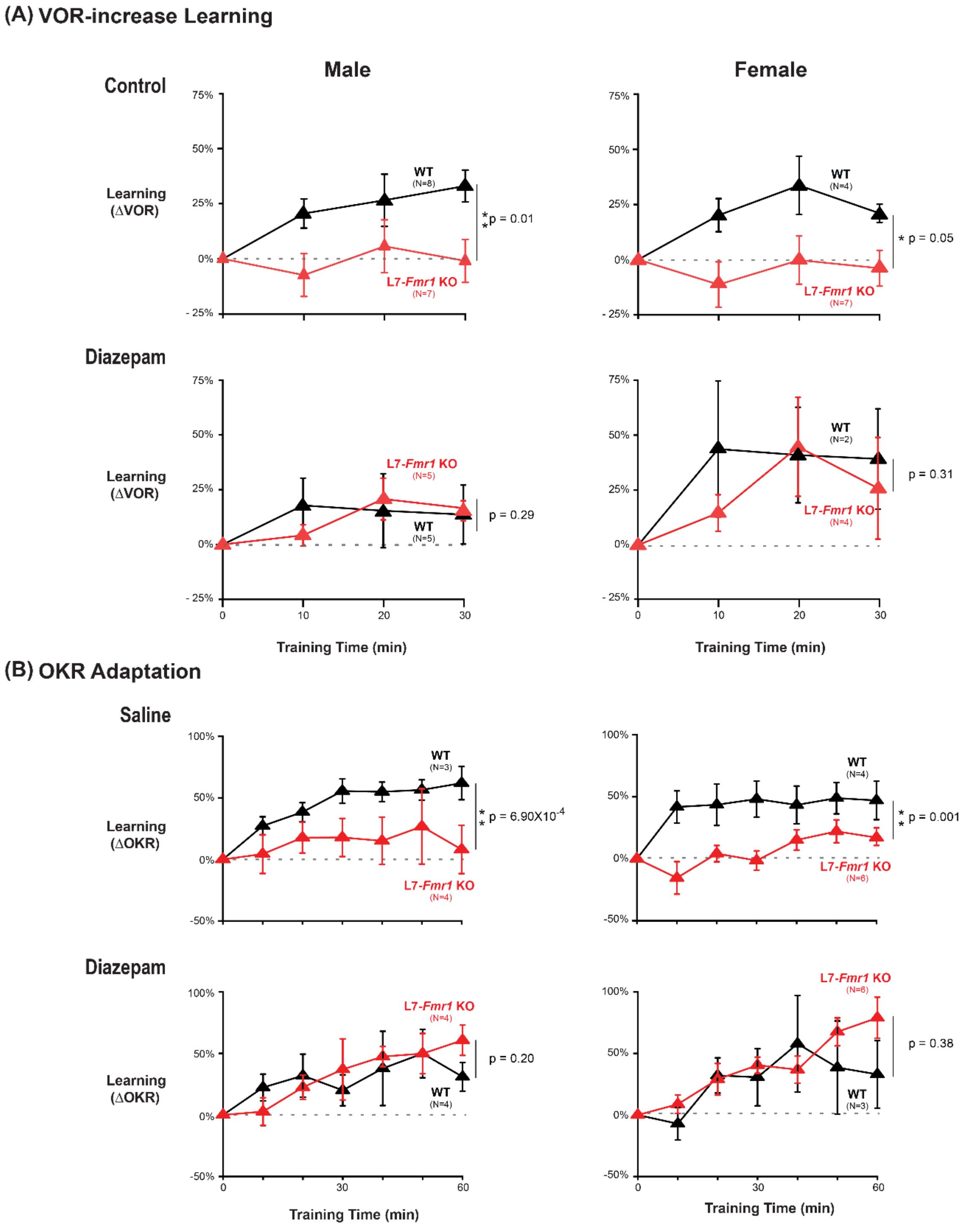

Figure 1 - figure supplement 1. Similar oculomotor learning impairments and efficacy of diazepam pretreatment in male and female L7-*Fmr1* KO mice. (A) VOR-increase training. ***Top Left,*** In male L7-*Fmr1* KO mice (*red*), VOR-increase learning was impaired relative to male WT (*black*) (p=0.01, 30 min, Tukey). ***Top Right,*** In female L7-*Fmr1* KO mice (*red*), VOR-increase learning was impaired relative to female WT (*black*) (p=0.05, 30 min, Tukey). ***Bottom Left,*** 18-24 hours after diazepam administration, male L7-*Fmr1* KO mice exhibited VOR-increase learning indistinguishable from that of male WT (p=0.290, 30 min, Tukey). ***Bottom Right***, 18-24 hours after diazepam administration, female L7-*Fmr1* KO mice exhibited VOR-increase learning indistinguishable from that of female WT (p=0.31, 30 min, Tukey). **(B)** OKR adaptation training. ***Top Left,*** In male L7-*Fmr1* KO mice, OKR adaptation was impaired relative to male WT ($p=6.90 \times 10^{-4}$, 30 min, Tukey). ***Top Right,*** In female L7-

bioRxiv preprint doi: https://doi.org/10.1101/2023.10.05.561013; this version posted April 5, 2024. The copyright holder for this preprint (which was not certified by peer review) is the author/funder, who has granted bioRxiv a license to display the preprint in perpetuity. It is made available under aCC-BY 4.0 International license.

Fmr1 KO mice, OKR adaptation was impaired relative to female WT (p=0.001, 30 min, Tukey). ***Bottom Left,*** 18-24 hours after diazepam administration, male L7-*Fmr1* KO mice exhibited OKR adaptation indistinguishable from that of male WT (p=0.20, 30 min, Tukey). ***Bottom Right***, 18-24 hours after diazepam administration, female L7-*Fmr1* KO mice exhibited OKR adaptation indistinguishable from that of female WT (p=0.38, 30 min, Tukey).

bioRxiv preprint doi: https://doi.org/10.1101/2023.10.05.561013; this version posted April 5, 2024. The copyright holder for this preprint (which was not certified by peer review) is the author/funder, who has granted bioRxiv a license to display the preprint in perpetuity. It is made available under aCC-BY 4.0 International license.

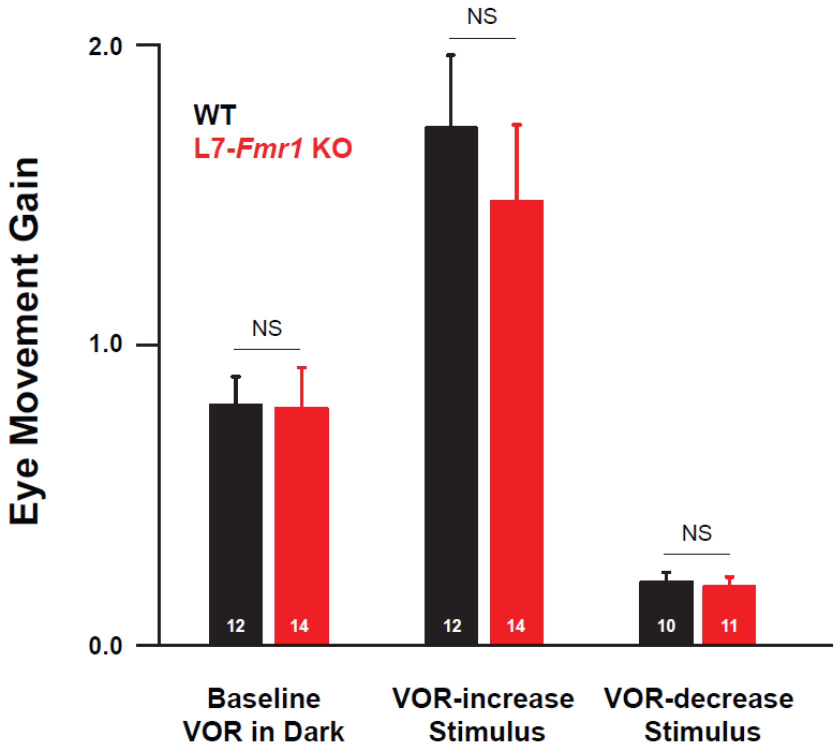

Figure 1-figure supplement 2. Baseline oculomotor performance of L7-*Fmr1* KO mice was indistinguishable from WT. The gain of the eye movement responses (ratio of eye movement amplitude to vestibular stimulus amplitude; see Methods) of L7-*Fmr1* KO mice (*red*) was not significantly different from that of WT mice (*black*) during baseline tests of the VOR in the dark before training (***left***; p= 0.95, two sample t-test) or during the first 45 sec of the paired presentation of visual and vestibular stimuli used for VOR-increase training (***middle***; p= 0.50, two sample t-test) or for VOR-decrease training (***right***; p= 0.76, two sample t-test). Number of mice tested is indicated in each bar.

bioRxiv preprint doi: https://doi.org/10.1101/2023.10.05.561013; this version posted April 5, 2024. The copyright holder for this preprint (which was not certified by peer review) is the author/funder, who has granted bioRxiv a license to display the preprint in perpetuity. It is made available under aCC-BY 4.0 International license.

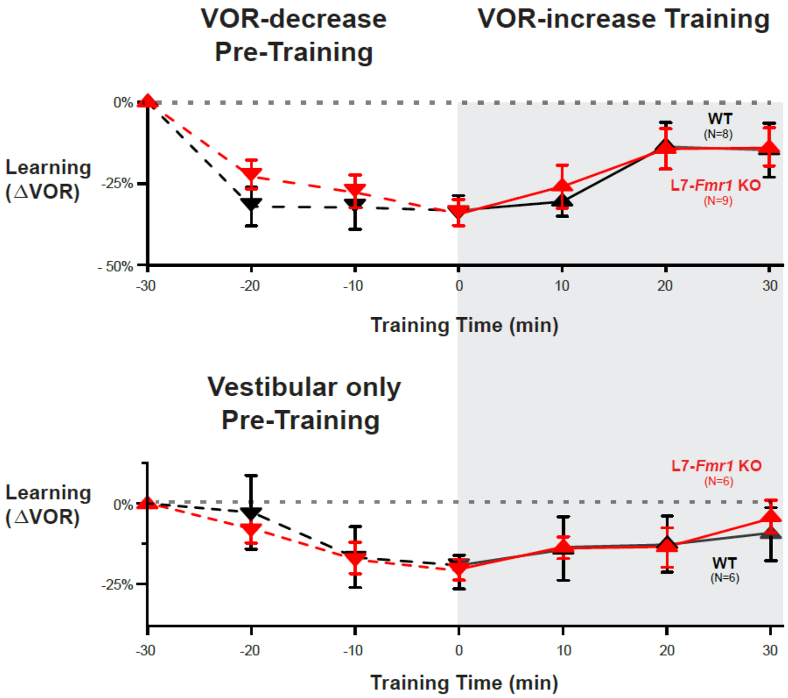

Figure 2-figure supplement 1. Data from Figure 2 were subsampled to compare VOR-increase learning in subpopulations of mice matched for the mean learned decrease in the VOR during pre-training. Subsampling was done by eliminating the WT mice (*black*) with the smallest decrease and L7-*Fmr1* KO mice (*red*) with the largest decrease in the VOR measured after 30 min of pre-training (just before the start of VOR-increase training), until the mean values in the two populations were within 2%. In these sub-sampled populations, the amount of VOR-increase learning was not significantly different between the L7-*Fmr1* KO and WT mice after VOR-decrease pre-training (*top;* p=0.74, L7-*Fmr1* KO mice vs. WT, 30 min, Tukey) or after Vestibular only pre-training (*bottom;* p=0.40, L7-*Fmr1* KO mice vs. WT, 30 min, Tukey), as also observed in the full samples.

bioRxiv preprint doi: https://doi.org/10.1101/2023.10.05.561013; this version posted April 5, 2024. The copyright holder for this preprint (which was not certified by peer review) is the author/funder, who has granted bioRxiv a license to display the preprint in perpetuity. It is made available under aCC-BY 4.0 International license.

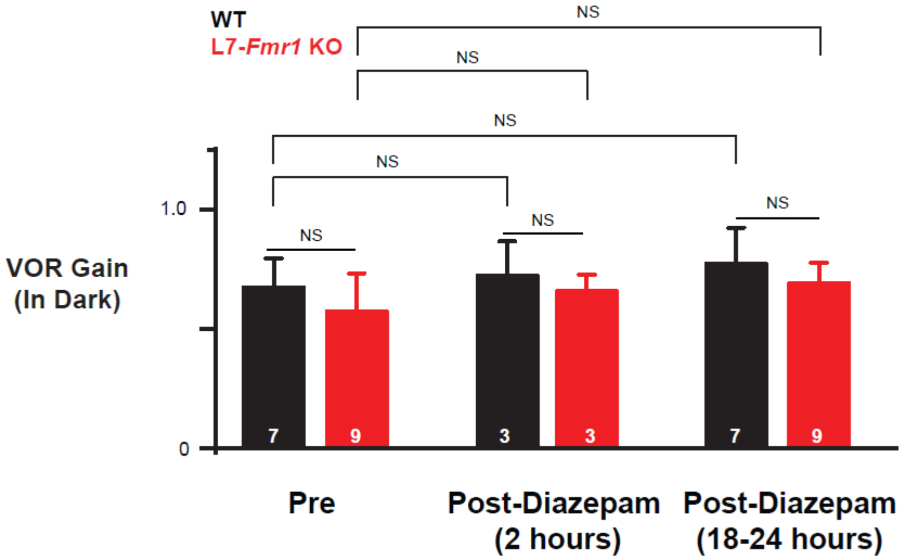

Figure 3 - figure supplement 1. Diazepam did not affect baseline VOR performance. The gain of the VOR (ratio of eye velocity to vestibular stimulus velocity) was measured in the dark in L7-*Fmr1* KO (*red*) and WT (*black*) mice before (*Pre*), 2 hours after (*Post-diazepam (2 hours)*) and 18-24 hours after (*Post-diazepam (18-24 hours)*) an IP injection of diazepam (0.5 mg/kg). There was no effect of diazepam on the gain of the VOR in L7-*Fmr1* KO mice (*red;* p=0.72, Pre vs. 2 hours Post Diazepam; p= 0.77, Pre vs. 18-24 hours Post-diazepam; Tukey) or WT mice (*black*; p=0.99, Pre vs. 2 hours Post diazepam; p= 0.36, Pre vs. 18-24 hours Post-diazepam; Tukey). Moreover, the gain of the VOR of L7-*Fmr1* KO mice was not significantly different from that of WT mice during baseline tests of the VOR in the dark before diazepam administration Pre (***left***; p= 0.79, Tukey), Post-diazepam (2 hours) (***middle***; p= 0.77, Tukey) and Post-diazepam (18-24 hours) (***right***; p= 0.97, Tukey). The 2-hour and 18-24-hour VOR performance measurements were made just before the VOR-increase training sessions (training time = 0) shown in **Fig. 3-figure supplement 2B**, and **Fig. 3B *top*,** respectively**.** The Pre VOR-performance measurements were made just before the VOR-increase training sessions shown in **Fig. 1A, *right*** for the subset of mice that were also tested 1 day after diazepam administration.

bioRxiv preprint doi: https://doi.org/10.1101/2023.10.05.561013; this version posted April 5, 2024. The copyright holder for this preprint (which was not certified by peer review) is the author/funder, who has granted bioRxiv a license to display the preprint in perpetuity. It is made available under aCC-BY 4.0 International license.

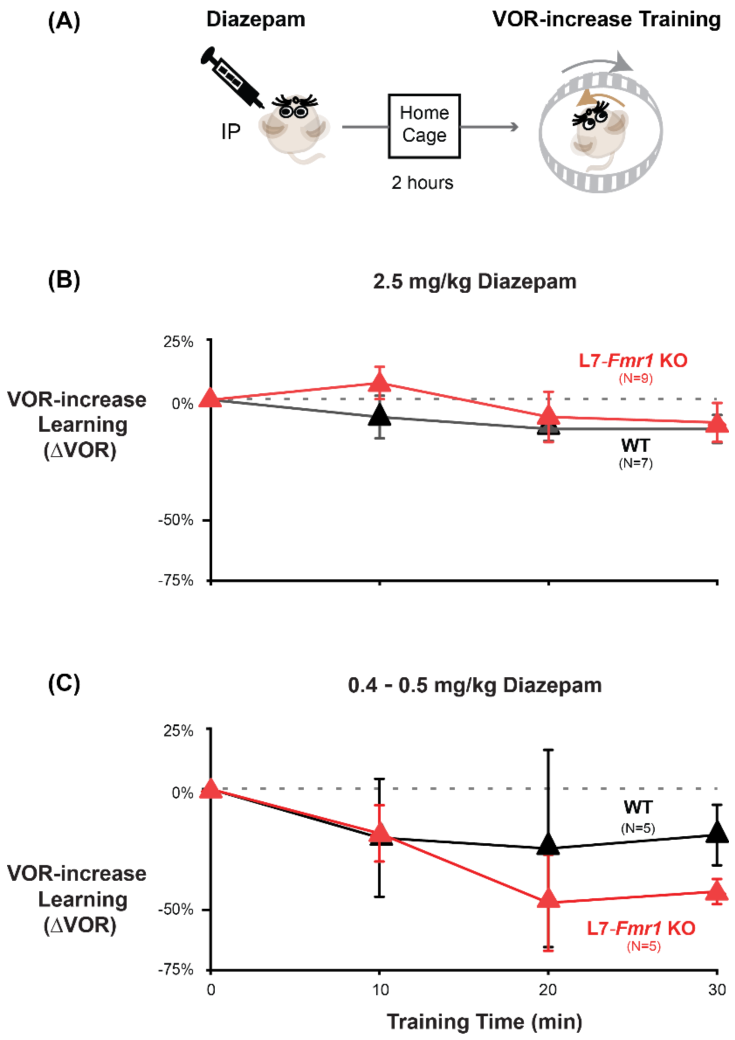

840

841 **Figure 3 - figure supplement 2.** The acute effect of diazepam was inhibition of VOR-increase learning. **(A)** Mice were given an
842 intraperitoneal (IP) injection of diazepam (2.5 mg/kg or 0.4-0.5 mg/kg) and then returned to the home cage for 2 hours before
843 VOR-increase training. When VOR-increase training was delivered two hours after IP injection of 2.5 mg/kg diazepam **(B)**, 0.4-
844 0.5 mg/kg diazepam **(C)**, no learned increase in VOR amplitude was observed in L7-*Fmr1* KO (*red*) or WT (*black*) mice.

bioRxiv preprint doi: https://doi.org/10.1101/2023.10.05.561013; this version posted April 5, 2024. The copyright holder for this preprint (which was not certified by peer review) is the author/funder, who has granted bioRxiv a license to display the preprint in perpetuity. It is made available under aCC-BY 4.0 International license.

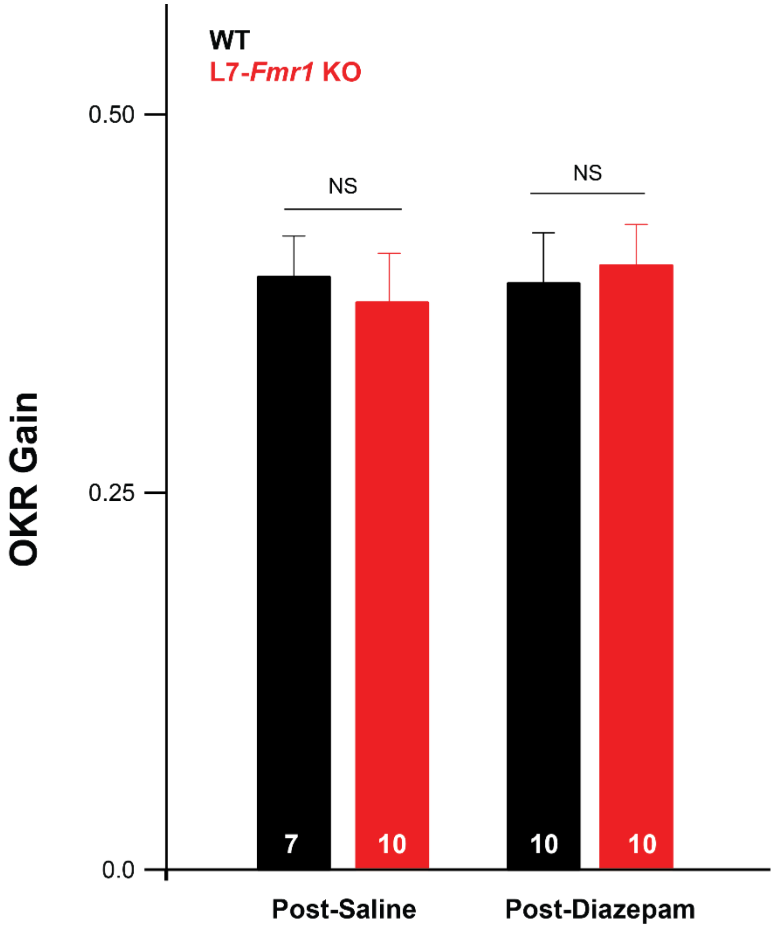

Figure 5 - figure supplement 1. Baseline optokinetic reflex (OKR) performance normal in L7-*Fmr1* KO mice and after diazepam pre-treatment. The baseline OKR was measured during the first three minutes of OKR adaptation training in L7-*Fmr1* KO (*red*) and WT (*black*) mice, 18-24 hours after an IP injection of saline or diazepam (0.5 mg/kg). There was no difference in the baseline OKR gain (ratio of eye velocity to optokinetic drum velocity) of L7-*Fmr1* KO vs. WT mice (p=0.690 Pre) and no effect of diazepam pre-treatment on the baseline OKR performance (L7-*Fmr1* KO, p= 0.690, post-saline vs. post-diazepam; WT, p= 0.55, post-saline vs. post-diazepam; Tukey).
