# Supplementary material for: Systemic pharmacological suppression of neural activity reverses learning impairment in a mouse model of Fragile X syndrome
Source: bioRxiv. 2023 Oct 5:2023.10.05.561013. Preprint. [Version 1] doi: 10.1101/2023.10.05.561013 (PMC10592955; doi:10.1101/2023.10.05.561013)
Supplement: Supplementary file 1 [file NIHPP2023.10.05.561013v1-supplement-1.pdf]

bioRxiv preprint doi: https://doi.org/10.1101/2023.10.05.561013; this version posted October 5, 2023. The copyright holder for this preprint (which was not certified by peer review) is the author/funder, who has granted bioRxiv a license to display the preprint in perpetuity. It is made available under aCC-BY 4.0 International license.

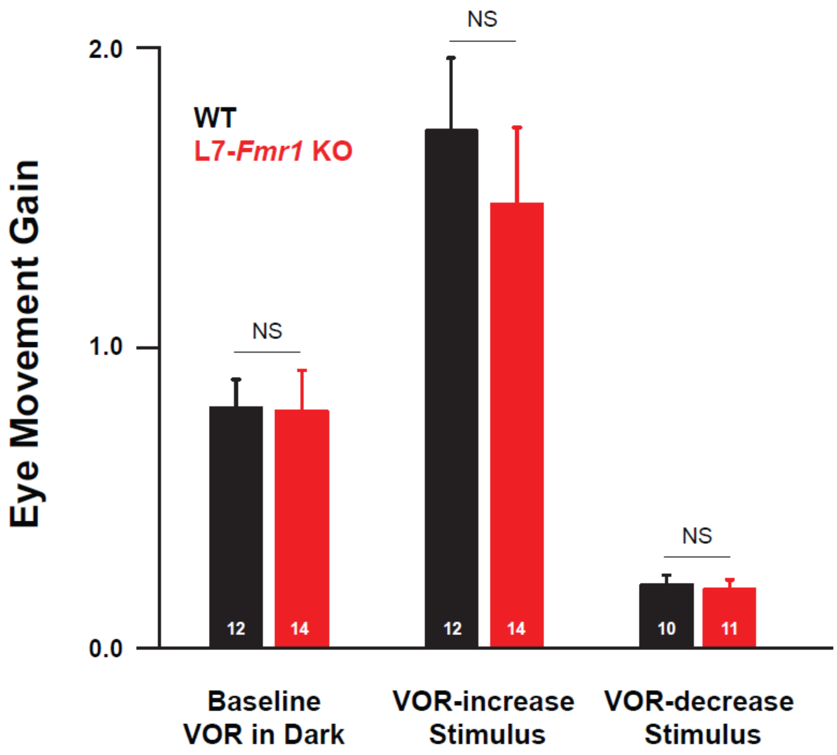

Figure 1-figure supplement 1. Baseline oculomotor performance of L7-*Fmr1* KO mice was indistinguishable from WT. The gain of the eye movement responses (ratio of eye movement amplitude to vestibular stimulus amplitude; see Methods) of L7-*Fmr1* KO mice (*red*) was not significantly different from that of WT mice (*black*) during baseline tests of the VOR in the dark before training (***left***; p= 0.95, two sample t-test) or during the first 45 sec of the paired presentation of visual and vestibular stimuli used for VOR-increase training (***middle***; p= 0.50, two sample t-test) or for VOR-decrease training (***right***; p= 0.76, two sample t-test). Number of mice tested is indicated in each bar.

bioRxiv preprint doi: https://doi.org/10.1101/2023.10.05.561013; this version posted October 5, 2023. The copyright holder for this preprint (which was not certified by peer review) is the author/funder, who has granted bioRxiv a license to display the preprint in perpetuity. It is made available under aCC-BY 4.0 International license.

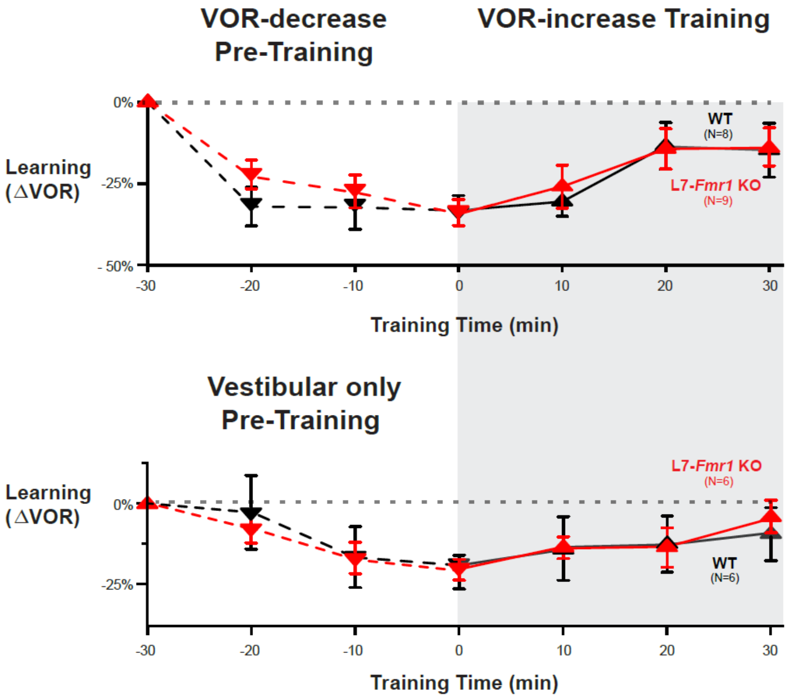

Figure 2-figure supplement 1. Data from Figure 2 were subsampled to compare VOR-increase learning in subpopulations of mice matched for the mean learned decrease in the VOR during pre-training. Subsampling was done by eliminating the WT mice (*black*) with the smallest decrease and L7-*Fmr1* KO mice (*red*) with the largest decrease in the VOR measured after 30 min of pre-training (just before the start of VOR-increase training), until the mean values in the two populations were within 2%. In these sub-sampled populations, the amount of VOR-increase learning was not significantly different between the L7-*Fmr1* KO and WT mice after VOR-decrease pre-training (*top;* p=0.74, L7-*Fmr1* KO mice vs. WT, 30 min, Tukey) or after Vestibular only pre-training (*bottom;* p=0.40, L7-*Fmr1* KO mice vs. WT, 30 min, Tukey), as also observed in the full samples.

bioRxiv preprint doi: https://doi.org/10.1101/2023.10.05.561013; this version posted October 5, 2023. The copyright holder for this preprint (which was not certified by peer review) is the author/funder, who has granted bioRxiv a license to display the preprint in perpetuity. It is made available under aCC-BY 4.0 International license.

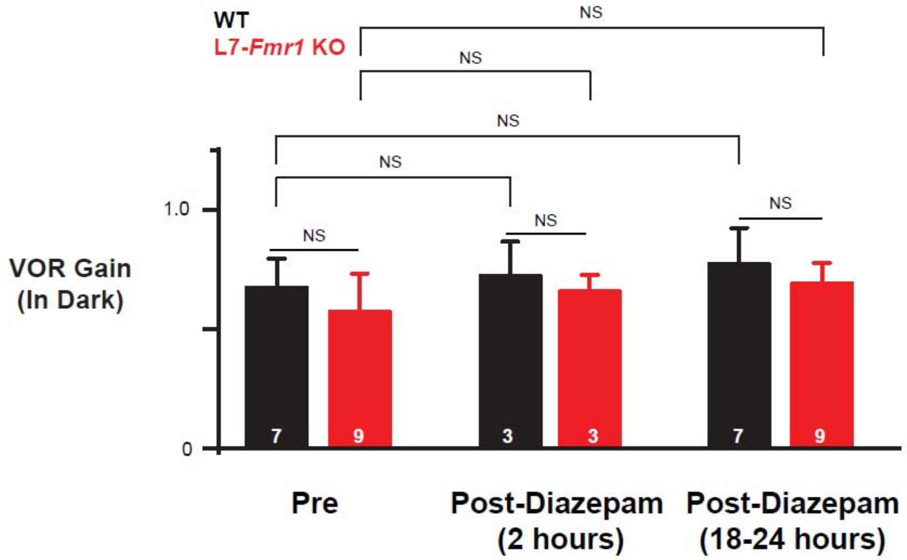

Figure 3 - figure supplement 1. Diazepam did not affect baseline VOR performance. The gain of the VOR (ratio of eye velocity to vestibular stimulus velocity) was measured in the dark in L7-*Fmr1* KO (*red*) and WT (*black*) mice before (*Pre*), 2 hours after (*Post-Diazepam (2 hours)*) and 18-24 hours after (*Post-Diazepam (18-24 hours)*) an IP injection of diazepam (0.5 mg/kg). There was no effect of diazepam on the gain of the VOR in L7-Fmr1 KO mice (*red;* p=0.72, Pre vs 2 hours Post Diazepam; p= 0.77, Pre vs 18-14 hours Post-Diazepam; Tukey) or WT mice (*black*; p=0.99, Pre vs 2 hours Post Diazepam; p= 0.36, Pre vs 18-14 hours Post-Diazepam; Tukey). Moreover, the gain of the VOR of L7-*Fmr1* KO mice was not significantly different from that of WT mice during baseline tests of the VOR in the dark before diazepam administration Pre (***left***; p= 0.79, Tukey), ***Post-Diazepam (2 hours)*** (***middle***; p= 0.77, Tukey) and ***Post-Diazepam (18-24 hours)*** (***right***; p= 0.97, Tukey). The 2-hour and 18-24-hour VOR performance measurements were made just before the VOR-increase training sessions (training time = 0) shown in **Fig. 3-figure supplement 2B**, and **Fig. 3B** ***top*****,** respectively**.** The Pre VOR-performance measurements were made just before the VOR-increase training sessions shown in **Fig. 1A,** ***right*** for the subset of mice that were also tested 1 day after diazepam administration.

bioRxiv preprint doi: https://doi.org/10.1101/2023.10.05.561013; this version posted October 5, 2023. The copyright holder for this preprint (which was not certified by peer review) is the author/funder, who has granted bioRxiv a license to display the preprint in perpetuity. It is made available under aCC-BY 4.0 International license.

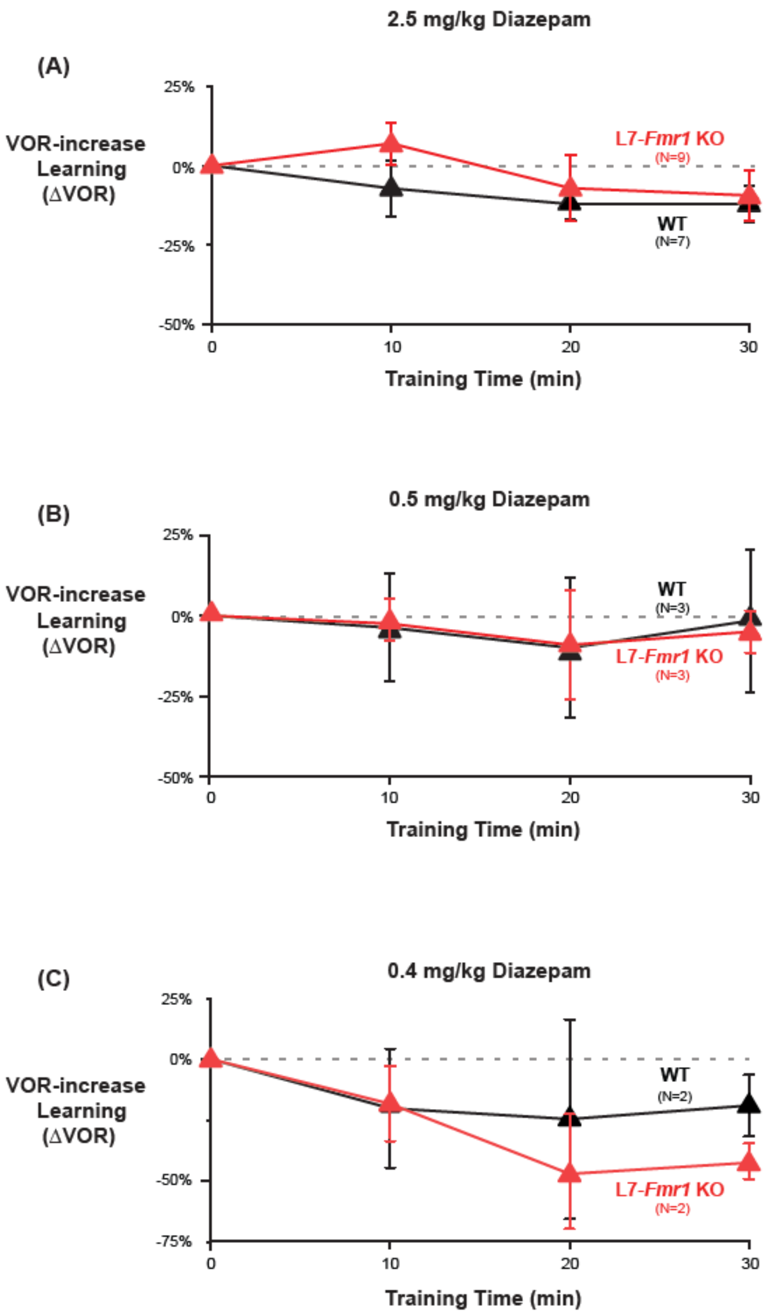

Figure 3 - figure supplement 2. The acute effect of diazepam was inhibition of VOR-increase learning. When VOR-increase training was delivered two hours after IP injection of 0.5 mg/kg diazepam **(A)**, 0.4 mg/kg diazepam **(B)**, or 2.5 mg/kg diazepam **(C)**, no learned increase in VOR amplitude was observed in L7-*Fmr1* KO or WT mice.
